# School-based healthy eating interventions for adolescents aged 10–19 years: an umbrella review

**DOI:** 10.1186/s12966-024-01668-6

**Published:** 2024-10-14

**Authors:** Nandeeta Samad, Lindsay Bearne, Farha Musharrat Noor, Fahmida Akter, Divya Parmar

**Affiliations:** 1 Department of Population Health Sciences, School of Life Course and Population Sciences, King’s College London, London, UK; 2 Population Health Research Institute, St George’s, University of London, London, UK; 3 James P Grant School of Public Health, BRAC University, Dhaka, Bangladesh

**Keywords:** Review, Healthy eating, School, Adolescents

## Abstract

**Background:**

The benefits of healthy eating are well known, yet adolescent diet is often poor. School based interventions offer a promising option to promote healthy eating, however, evidence is unclear.

**Aim:**

This umbrella review synthesised the current evidence on school-based interventions for healthy eating in adolescents (10–19 years old).

**Methods:**

Using Joanna Briggs Institute (JBI) umbrella review guidelines, a systematic search was conducted on 11 electronic databases (PubMed, CINHAL, EMBASE, Science Direct, PsycINFO, MEDLINE, Scopus, ERIC, Web of Science, Cochrane Register of Systemic Review and JBI Evidence Synthesis) to identify reviews published between January 2000 and December 2023. Methodological quality was assessed using JBI critical appraisal tool. A narrative synthesis was conducted informed by the World Health Organisation’s Health Promoting School (HPS) framework that categorises school-based interventions into three components i.e., health education, school environment changes, and family and community involvement.

**Results:**

Seventeen reviews were identified (including 347 unique primary studies) that were published between 2008 and 2023. 87% of the reviews were based on interventions in high- income countries, limiting applicability to low- and middle-income countries. Fourteen reviews were rated as high, two as moderate, and one was rated as low methodological quality. Evidence from 71% of the reviews (*n* = 14 reviews, 13 = high methodological quality) found that multi-component interventions (i.e., interventions incorporating more than two components of the HPS framework) improved adolescents’ knowledge and behaviour concerning healthy eating. At the individual level, tech-driven healthy eating curricula effectively improved eating behaviours of adolescents. These individual-level interventions proved to be more effective and sustainable when supported by system-level changes, such as modifying school environments including increased availability of healthy foods and involving parents to promote healthy eating for adolescents. However, limited evidence from only three reviews suggests mixed feasibility for technology-based interventions and lower feasibility for multi-component interventions. The lack of information on stakeholder involvement in intervention design is another critical evidence gap.

**Conclusion:**

School-based multi-component healthy eating interventions that combine individual-level interventions with system-level changes are effective in promoting healthy eating behaviours among adolescents. Future reviews should assess the effectiveness of participatory approaches in intervention design, feasibility and scale-up studies, and analysing evidence from low- and middle-income countries.

**Supplementary Information:**

The online version contains supplementary material available at 10.1186/s12966-024-01668-6.

## Introduction

Healthy eating is essential for adolescents’ physical and mental development, providing the calories and nutrients needed to support their growth, development, and the maintenance of an active lifestyle throughout their lives [1, 2]. Unhealthy eating contributes to obesity and associated health issues among adolescents such as growth retardation, impaired organ development, micronutrient deficiencies, and later in life can lead to non-communicable diseases (NCDs) including cardiovascular diseases, diabetes mellitus, and hypertension [3–7]. Adolescents (aged between 10 and 19 years old) [8] need to consume a daily intake of 2200 to 3000 calories, with a balanced distribution of macronutrients, including carbohydrates (45–65% of total energy intake), protein-rich foods, such as fish and meat (10–30%), and fats (25–35%) [9, 10]. Diets should also include at least five servings of fruits and vegetables (FV) rich in vitamins, minerals and fibre, 2.5-3 servings of dairy products and limit the intake of added sugar (less than 10% of total energy intake) and high fat foods [9, 10].

Adolescents in both low- and middle-income countries (LMICs) and high-income countries (HICs) frequently have diets that are calorie-dense yet nutrient-deficient, marked by excessive consumption of sugar-sweetened beverage (SSB), ultra-processed foods, and insufficient intake of FV [11–13]. Ultra-processed foods are laden with added sugars, salt and harmful fats, and are deficient in essential nutrients like dietary fibre, vitamins, and minerals [14]. These should be avoided as they pose significant health risks including increased risk of cardio-metabolic events [14]. A meta-analysis examining the Global School-based Student Health Surveys from 2008 to 2015 including Africa, Asia, Oceania, and Latin America revealed that 35% of adolescents do not meet the recommended intake of FV, 43% consume sugary sweetened beverages (SSBs) daily, and 46% eat processed foods at least weekly [12]. Furthermore, a recent UNICEF report drew attention to the low FV intake among adolescents worldwide [13]. The prevalence of meal skipping among adolescents, especially breakfast, has also been linked to increased fast food consumption [15, 16].

School can play a critical role in promoting healthy eating among adolescents. Broadly speaking, school-based healthy eating interventions use two approaches: individual-level interventions, which tailor curricula to influence adolescents’ behaviours, and system-level interventions, which embed strategic actions into daily life to modify school policies [17]. The World Health Organisation’s (WHO) Health Promoting School (HPS) framework [18] provides a comprehensive approach to promoting healthy eating in schools, encompassing three key components: health education, school environment modifications, and engagement with families and communities. However, despite there being a plethora of school-based intervention, including many reviews, there is a lack of synthesised evidence on the diverse components and contents of these interventions and their impact on adolescents’ eating behaviour. The existing literature has not adequately explored the effectiveness of specific intervention strategies within each component of HPS. One umbrella review assessed school-based healthy eating interventions focusing on behaviour changes in children aged 6 to 18 years, it did not present results separately for adolescents [19]. This is important as adolescents have unique developmental needs and challenges that require tailored intervention approaches. The lack of adolescent-specific evidence limits the ability to design and implement interventions that effectively address the unique barriers and facilitators to healthy eating in this age group. Moreover, the umbrella review’s omission of a synthesis of the interventions’ specific components and their respective contents constitutes a notable evidence gap that merits further exploration [19]. The comprehensive synthesis of intervention components and their respective contents is crucial for understanding the effectiveness, generalisability, and replicability of these interventions [20]. This umbrella review addresses these evidence gaps by synthesising evidence from reviews evaluating school-based healthy eating interventions targeting adolescents. This review will provide insights to inform the development and implementation of evidence-based, tailored interventions that promote sustainable healthy eating among adolescents in school settings.

## Materials and methods

This umbrella review followed the Joanna Briggs Institute’s (JBI) methodology for umbrella reviews [21] and is reported in accordance with the Preferred Reporting Items for Overviews of Reviews (PRIOR) [22] (Supplementary file 1). The umbrella review protocol is registered with the PROSPERO database for systematic reviews (CRD42022338762).

### Eligibility criteria

Our population of interest were adolescents aged 10 to 19 years. Reviews on broader age range were included if they reported data for adolescents separately. School-based interventions promoting healthy eating were included, and interventions promoting other healthy behaviours such as physical activity were included only if outcomes related to healthy eating were reported separately. Comparison groups included no intervention, or comparison to one or more other interventions. Reviews using standardised measures, such as changes in healthy eating knowledge and behaviours among adolescents, were included and those that reported non-dietary or non-nutritional outcomes such as obesity, unhealthy weight, anthropometric measurements, BMI, metabolic outcomes, and physical activity, were excluded. Reviews were selected if they reported both dietary and non-dietary outcomes separately, based on specific primary studies included in their analysis. This criterion ensured that reviews providing distinct information on outcomes regarding healthy eating knowledge and behaviour were included in our study. All types of reviews were included- systematic reviews with or without meta-analyses, narrative reviews, scoping reviews, rapid reviews, critical reviews, and integrative reviews. Peer-reviewed published reviews were considered, while protocols, conference abstracts and proceedings, commentaries, editorials, unpublished reviews, or reviews published as grey literature were excluded. We included reviews published between 1st January 2000 and 31 December 2023 and written in English.

### Search strategy

#### Database search

Eleven electronic databases were searched: PubMed, Cumulated Index to Nursing and Allied Health Literature (CINHAL), Excerpta Medica dataBASE (EMBASE), Science Direct, Psychological Information Database (PsycINFO), Medical Literature Analysis and Retrieval System Online (MEDLINE), Scopus, Education Resources Information Center (ERIC), and Web of Science, Cochrane Register of Systemic Review, and JBI Evidence Synthesis.

#### Search terms

Keywords for school-based interventions and healthy eating were discussed among the research team and further refined by consulting with a senior librarian at King’s College London. The search strategy was then piloted in PsycINFO, via Ovid, and Scopus before search terms were finalised (Supplementary file 2).

### Review screening

Records identified from database search were exported to Rayyan [23]. After removing duplicates, titles and then abstracts were reviewed against the eligibility criteria by two independent reviewers (NS and FN). Full texts of eligible records were reviewed independently by NS and FN. The reasons for exclusion were recorded. Any discrepancies were resolved by consensus between the two reviewers and when required, a third reviewer (LB or DP or FA) was consulted. To assess the extent of overlap between reviews, we created a citation matrix following Cochrane guidelines [24] (Supplementary file 3). We included all relevant reviews in our study, even if they shared some primary studies. However, we found no instances where one review completely overlapped with another in terms of primary studies.

### Quality appraisal

The included reviews were appraised using the standard JBI critical appraisal tool by two independent reviewers (NS and FN). Seventy per cent of these were checked by another researcher (LB or DP). The tool consisted of 11 questions (responses: “Yes”, “No”, “Unclear” or “NA”). The overall score of a review was calculated by summing the affirmative answers (range 0–11 points). This tool does not mention cut-off points for categorising the quality of systematic reviews [21], hence, we applied these cut-off points: high quality (≥ 8 “Yes”), moderate quality (5–7 “Yes”), and low quality (≤ 4 “Yes”) (Supplementary file 4).

### Data extraction

We extracted the following data from the included reviews: author and date, publication year, type of review, total number of included studies, age groups of the study participants, countries of the primary studies, study designs, studied interventions (components, contents, duration), outcome, and key findings. Data were extracted independently by two researchers (NS and FN), and 70% of the extracted data was checked by a third researcher (LB or DP). We adopted the Template for Intervention Description and Replication (TIDieR) framework to identify the intervention components, i.e., distinct element of the overall intervention strategy, and intervention ‘content’, i.e., specific materials, procedures, activities, and information that are provided or used within each component of the intervention [25].

### Data synthesis

We conducted a narrative synthesis of the finding [26]. We categorised the intervention components according to the World Health Organisation’s (WHO) Health Promoting School (HPS) framework and interventions with two or more components were categorised as multi-component interventions [18]. We reported findings on the effectiveness of eating knowledge and behaviour outcomes according to a framework previously employed in a Cochrane overview of reviews framework [19, 27]. This framework evaluates the effectiveness of interventions as [19, 27]: “Likely effective” if evidence supporting intervention effectiveness is based on meta-analysis or narrative synthesis of all primary studies; “Promising” if evidence of effectiveness is based on over 50% of primary studies but requires further confirmation; “Probably ineffective” if majority of the primary studies results are ineffective; “Ineffective” if findings in all primary studies are found to be ineffective; and “Inconclusive” if there is inadequate evidence on effectiveness. Additionally, we applied the Behaviour Change Technique Taxonomy (BCTT) to identify effective combinations of intervention components for promoting healthy eating behaviours among adolescents [28].

## Results

**Fig. 1:**
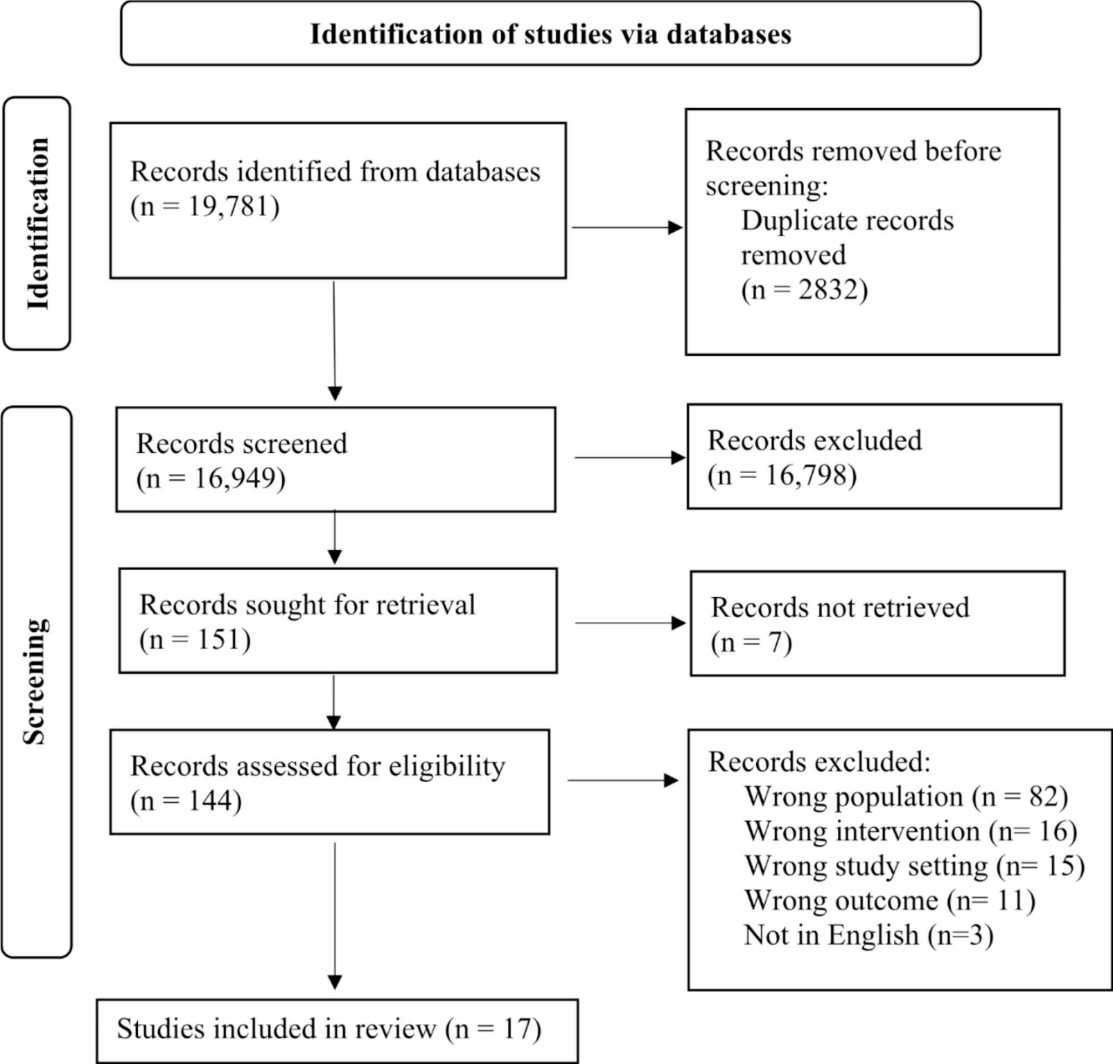
PRIOR flow diagram

A total of 19,781 records were identified through database searching (Fig. 1). After deduplication, 16,949 titles and abstracts were screened, and 151 reports were identified for full text screening. Out of 151 full text reports, four could not be retrieved because they were in conference proceedings. Remaining 144 full-text reports were assessed for eligibility, and 17 reviews were included in this umbrella review.

### Characteristics of included reviews

The 17 included reviews were published between 2008 and 2023 and included studies published between 1987 and 2020 (Table 1). A total of 347 unique primary studies were captured in these 17 reviews. Fifteen of the reviews used narrative synthesis [29–43] and two included both meta-analysis and narrative synthesis [44, 45]. Two reviews included primary studies with a broader age range, but synthesised results for adolescents separately [32, 34]. We incorporated these adolescent-specific findings in our synthesis. Majority of the reviews (*n* = 14) evaluated multi-component interventions [30–32, 34–40, 42–45] while three reviews evaluated only health education interventions [29, 33, 41]. All the reviews included studies based in HICs, and only eight reviews included a few studies based in five LMICs [30, 35–38, 42, 43, 45]. Several tools were used to measure the outcomes of the interventions. According to the JBI critical appraisal tool, 14 reviews scored high [30–32, 34–41, 43–45], two reviews scored moderate [33, 42], and only one review scored low [29] in terms of methodological quality (Table 2).

**Table 1:** Characteristics of the included reviews (*n* = 17)

Author; Year;	Type of reviews	Total number of unique primary studies included; publication period	Population Age range	Included countries	Outcomes	Outcome measures
Alcântara et al., 2018 [29]	Integrative review	8; 2004–2016	10–19 years	HICs: France, Italy, US	Knowledge about health eating, and intake of FV, processed snacks, and SSB	Survey
Bailey CJ et al., 2019 [36]	Systematic review	44; 1996–2016	10–19 years	HICs: Australia, Canada, China, Denmark, France, Greece, Northern Ireland, Norway, Portugal, South Africa, Sweden, UK, US LMICs: India, Iran, Kenya	Eating knowledge, FV, processed snacks intake,	FFQ, 24 h dietary recalls, interviews, focus groups, audio/video-taping, observations, surveys
Calvert S et al., 2019 [37]	Systematic review	29; 1987–2017	11–16 years	HICs: Australia, Belgium, Canada, China, Denmark, England, Greece, Israel, Netherlands, Norway, Spain, Taiwan, US LMIC: Tunisia	FV, processed snacks, SSB, calorie, fat, protein, fibre, vitamins, frequency of regular meal consumption,	FFQ, 24-h recall
Champion KE et al., 2019 [44]	Systematic review and meta-analysis	13^a^; 2003–2017	Mean: 13.41 years	HICS: Belgium, Mexico, Netherland, Spain, US	Eating knowledge, FV intake, fat, fibre, processed snacks, SSB,	Self-administered survey, FFQ, 24-h recall, 3-day food record
Hackman et al., 2014 [38]	Systematic review	11; 2005–2013	10–19 years	HICs: Australia, Canada, England, Greece, US, Scotland, South Africa LMIC: Iran	FV intake, processed snack, frequency of breakfast consumption, stay in school for lunch	24-h recall, cognitive and attitudinal assessments, food diary, FV recall, number of days stayed, bought, ate school for lunch, snack scale, FFQ
McHugh C et al., 2020 [39]	Systematic review	4^b^; 1998–2016	11–18 years	HICs: Finland, US	FV, fat	24-h recall, self-administered KAP survey
Medeiros et al., 2022 [45]	Systematic review and meta-analysis	24; 1997–2019	10–19 years	HICs: Belgium, Brazil, China, Ecuador, Finland, Greece, Italy, Netherland, Norway, Trinidad Tobago, UK, US LMIC: Iran	FV, processed snacks, intake	FFQ, 24-h recall, 7-day food survey, KAP
Meiklejohn et al.; 2016 [40]	Systematic review	13; 2002–2013	10–18 years	HICS: Australia, Belgium, Finland, Greece, Netherland, Norway, Spain, Sweden, US	FV, processed snacks, water, protein intake,	FFQ, 24-h recall
Melo GRDA e al., 2017 [41]	Systematic review	11; 2007–2015	10–17 years	HICs: Austria, Australia, Belgium, Denmark, Germany, Greece, Netherlands, Spain, Sweden, Taiwan, UK, US	Eating knowledge, FV, SSB, processed snacks, fat intake,	FFQ
Nakabayashi J et al., 2020 [42]	Systematic review	14; 2003–2019	10–17 years	HICS: Belgium, Brazil, England, Malaysia, Mexico, Spain, Turkey, US LMIC: Iran	FV, fat, calorie intake,	FFQ, 5-day recall, food diary
Pierre CS et al., 2021 [43]	Systematic review	53; 2005–2019	10–14 years	HICs: Aruba, Australia, Canada, China, New Zealand, US LMIC: Ethiopia	Eating knowledge, FV, SSB, frequency of breakfast consumption, willingness to try healthy foods	Surveys and focus groups
Rose K et al., 2021 [31]	Systematic review	27; 2009–2019	12–18 years	HICs: Denmark, France, Finland, Greece, Italy, Netherlands, Norway, Portugal, Spain, UK, Turkey	Eating knowledge, FV, processed snacks, SSB, calorie, water intake, frequency of meal consumption, food choice competency	survey, cashless system- transactions from point of sale/till, freestanding interactive computer terminals
Sa JD & Lock K, 2008 [32]	Systematic review	7^c^; 1999–2007	11–18 years	HICs: Belgium, Norway, US	FV intake	FFQ, 24 h recall, KAP
Shinde et al., 2023 [30]	Systematic review	27^d^; 2006–2020	10–19 years	HICs: Brazil, China, Malaysia, Palestine, Turkey LMICs: Ethiopia, India, Iran	Eating knowledge, FV, processed snacks, SSB, breakfast frequency	Not reported
Tallon JM et al., 2019 [33]	Systematic review	13; 2004–2018	12–18 years	HICs: Belgium, Denmark, UK, US	Eating knowledge, FV, fat, meal frequency	Not reported
Van Cauwenberghe et al., 2010 [34]	Systematic review	13^e^; 1991–2008	13–18 years	HICs: Belgium, Denmark, France, Italy, Netherlands, Norway, Sweden, UK	FV, fat, water, SSB, fish	Food diary, 24-h recall, FFQ, self-reported questionnaire, observation, sales data
Vézina-Im LA et al., 2017 [35]	Systematic review	36; 1989–2016	12–17 years	HICs: Australia, Belgium, Brazil, Canada, China, Korea, Netherlands, US LMICs: India	SSB consumption	FFQ, 24-h recall, web-based self-administered survey

HICs: High Income Countries; LMICs: Low- and middle-income countries; FV: Fruits and Vegetables; SSB: Sugar-sweetened Beverage; FFQ: Food Frequency Questionnaire; a: out of 22 unique primary studies assessing different health outcomes, 13 specifically reported on healthy eating outcomes; b: out of 12 unique primary studies assessing different health outcomes, four specifically reported on healthy eating outcomes; c: out of 30 unique primary studies assessing healthy eating outcomes for children and adolescents, seven reported on adolescents aged 11 to 18 years; d: out of 68 unique primary studies, 27 specifically reported on healthy eating outcomes; e: out of 42 unique primary studies assessing healthy eating outcomes for children and adolescents, 13 specifically reported on adolescents

### Single-component interventions

Out of 17 reviews, three reviews [29, 33, 41], comprising a total of 32 unique primary studies, focused on single-component individual-level interventions (Table 2). The methodological quality of these reviews was mixed: one study was rated as high [41], one as moderate [33], and one was rated as poor methodological quality [29]. These reviews exclusively synthesised data from HICs. All the three reviews focused on promoting health eating and included some tech-driven curriculum, i.e., the integration of technological tools into educational practices. The contents included lessons on nutrition, personal diet recommendations, gamified learning experiences (such as levelling up based on healthy eating knowledge and behaviour), cooking recipes, and an app to record daily food intake. Only one review [38] reported on the theoretical frameworks that underpinned interventions - the social cognitive theory (SCT), social learning theory (SLT), and theory of reasoned action (TRA). The intervention duration, ranged from two to 10 weeks [30, 38] and the timing of follow up assessments ranged from immediately after intervention to three years after the intervention [30, 38]. Primary outcomes for these reviews were healthy eating knowledge and behaviour such as consumption of FV, dairy, meat and fibre, tendency to skip meals and intake of processed snacks and SSBs [26, 30, 38]. Two reviews [26, 38] used the food frequency questionnaire (FFQ) to measure outcomes and the third review did not report such tools [30]. Applying the effectiveness categorisation framework [25], these interventions were considered “likely effective” in improving both knowledge about healthy eating and actual eating behaviours. Two reviews [26, 38] reported on acceptability of the tech-driven curriculum and reported there was higher participation and engagement by adolescents. These reviews also found that these interventions improved accessibility overall and were equitable as they were able to engage adolescents with low resources. Flexible participation, time-saving and the ability to customise content by language were key features that improved the feasibility of these interventions [26, 38]. The combination of three BCTT hierarchical clusters was “likely effective”, as reported by only one review with high methodological quality: feedback and monitoring (SMS-based diaries); shaping knowledge (computer-tailored workshops); and associations (SMS) (Supplementary file 5).

**Table 2:** Evidence on single component (healthy eating education) interventions

Author; Year	Intervention design of studies included in the review	Interventions description	Findings	Cochrane categorisation of effectiveness; JBI critical appraisal score
Alcântara et al., 2018 [29]	**Study design**: RCT (*n* = 5), quasi-experimental (*n* = 2), mixed-methods (*n* = 1) **Theories**: Not reported	**Components**: computer-tailored workshops, virtual canteen, blogs, games **Contents**: lessons on nutrition, personal healthy eating dietary recommendations, and gamified learning experiences, such as level up based on healthy eating knowledge, eating behaviour, intake measures **Duration of intervention**: Not reported **Follow-up range**: Not reported	All included reviews reported improved healthy eating knowledge, increased FV intake, decreased intake of processed snacks and SSBs	Likely effective; 4 (low quality)
Melo GRDA e al., 2017 [41]	**Study design**: RCT (*n* = 7), quasi-experimental (*n* = 4) **Theories**: SCT, SLT, TTM, TPB, TRA	**Components**: computer-tailored workshops,, SMS, SMS-based diaries **Contents**: nutritional lessons and dietary guidance, healthy cooking recipes via handbooks, guidance leaflets, and sending timed SMS for users to report food intake, real-time tracking and feedback on eating behaviour, as contents of SMS-based diaries **Duration of intervention**: 2–10 weeks **Follow-up range**: 2 weeks to 2 years	All the included studies reported improved healthy eating knowledge, increased intake of FV, dairy, meat, and fibre, decreased intake of processed snacks and SSB	Likely effective; 9 (high quality)
Tallon JM et al., 2019 [33]	**Study designs**: Not reported **Theories**: Not reported	**Components**: workshops, games, SMS-based diary, apps**Contents**: healthy eating knowledge and advice, app to to measure and monitor daily food intake **Duration range**: not reported **Follow-up range**: 1 month to 3 years	All included studies reported improved healthy eating knowledge and behaviour with increased FV, decreased fat intake, decreased meal skipping	Likely effective; 6 (moderate quality)

RCT: Randomised Control Trial; SCT: Social Cognitive Theory; SLT: Social Learning Theory; TTM: Transtheoretical Model; TPB: Theory of Planned Behaviour; TRA: Theory of Reasoned Action; SMS: short message service

### Multi-component interventions

Fourteen reviews, including 313 unique primary studies, assessed interventions with at least two components: healthy eating education, changes to the school environment, and family involvement (Table 3). Thirteen reviews were rated as high, and only one was rated as moderate methodological quality. These reviews mostly included studies based in HICs. Eight reviews among these 14 included primary studies based in five LMICs [30, 35–38, 42, 43, 45].

Two high-quality reviews (including 80 unique primary studies) found that interventions incorporating all three components of the HPS framework, were “likely effective” in improving healthy eating knowledge and behaviour, particularly increased consumption of FV and water, reduced consumption of SSB, total daily calories, regularly eating breakfast and other meals, willingness to try healthy foods, and improved food choice competency in HICs [31, 43]. One of these reviews included a single study from an LMIC, Ethiopia [43].

The **healthy eating education** components at the individual level, included lectures, tailored leaflets, handbooks, text messages, board games, drama, mobile health counselling, healthy eating club, and motivational visits from athletes and other role models. The contents involved healthy eating information, nutrition, healthy cooking lessons, club activities, such as healthy eating photography.

The **school environment change** components at the system level, included school-wide marketing and canteen modification with contents involving healthy food promotion and increased availability of healthy foods in schools. In the context of healthy eating interventions in our included reviews, both terms “canteen” and “cafeteria” refer to the main food service area in a school. We have used “canteen” consistently throughout.

The **family involvement** components at the system level, included parents’ meetings and homework with contents on healthy eating information and feeding healthy foods at home.

Only one of the reviews [43] commented on the theoretical models on which the interventions were based - trans-theoretical model (TTM), SCT, theory of planned behaviour (TPB), and attitudes social influence self-efficacy (ASE) model. Interventions in studies in these reviews ranged from one to 18 months. The outcomes were measured by surveys, focus groups, or sales transactions [31, 43]. Components related to the school environment, such as increased availability of healthy foods and parental involvement requires resources for implementation and so were found to be less feasible [31].

There were promising effects of multi-component interventions on healthy eating behaviour reported in seven high-quality [30, 32, 35–38, 45] and one moderate-quality reviews [42] (including 192 unique studies) [30, 32, 35–38, 42, 45]. The primary outcomes for these reviews were intake of FV [30, 32, 36–38, 42, 45], processed snacks [30, 36–38, 45], SSB [30, 35, 37], fat [37, 42], protein, fibre and vitamins [37], frequency of regular meals [30, 37, 38], recommended calorie intake [37, 42], healthy eating knowledge [30, 36], and staying in school for having healthy lunch [38]. These reviews mainly focused on interventions in HICs - only 11 primary studies were from LMICs (Ethiopia, India, Iran, Kenya, and Tunisia) [30, 35–38, 42, 45]. One review (including 14 unique studies) included healthy eating education and family involvement [42], one review (including 44 unique studies) included healthy eating education and environmental changes [36], and rest six reviews (including 134 unique studies) included all the three components of the HPS framework [30, 32, 35, 37, 38, 45].

The **healthy eating education** components at the individual level, included lectures [30, 32, 36, 38, 42, 45], quizzes and games [30, 36, 38], media shows [37], plays, electronic messages, rewards, peer-leading activities and training for teachers [38]. The contents involved lessons on healthy eating [32, 37, 38, 42, 45], food labelling, healthy cooking [30, 36], consequences of SSB intake [35], nutrition, food safety farmers’ visits on healthy food cultivation [36], menu planning, healthy eating goal setting and self-monitoring, healthy food as rewards, and food tasting [38].

The **school environment change** components at the system level, involved FV gardening [32, 36], school food marketing [38, 45], canteen modifications [30, 36], vending machine modifications [35], postering [45], workshops with kitchen staff [36], and loyalty programs [32]. The contents involved increased availability of milk and protein [32, 35–38, 45] and fresh fruits [30], free or subsidised FV [35], replacing SSB with healthier alternatives (milk, water, juice) in vending machines [35], chef, staff consultations on healthy culinary lessons [36], and healthy eating posters around school [45].

The **family involvement** components at the system level included communicating with families via newsletters [30, 32, 35, 37, 38], leaflets [30, 45], emails [35, 37], booklets [30], brochures [30] and texts [37], organising parents’ meetings [35, 45], in-school learning sessions, food coupons [37] and social support groups [35], and providing parents recipe guides [30]. The contents involved information on healthy eating knowledge [30, 32, 35, 38, 42, 45], healthy cooking, feeding healthy foods to children at home [37], nutritional guidelines [38], coupons to purchase healthy foods [37], offering FV and free healthy foods [45].

The interventions within the studies were informed by several theoretical models - SCT [30, 35, 36, 45], TPB [30, 35, 38, 45], TTM [36, 42, 45], health belief model [30, 36], TRA [30, 38], pedagogy of the oppressed by Paulo Freire [30, 45], socio-ecological model (SEM) [30, 45], self-regulation theory (SRT) [35, 45], ASE Model, the action planning literature, Bloom’s mastery of learning model, Bronfenbrenner’s ecological theory [45], cognitive behavioural theory [30], health action process approach [30], HPS [30], diffusion of innovation theory (DIT) [40], expectancy theory [40], self-determination theory [40], elaboration likelihood model [35], and theory of interpersonal behaviour [35]. The outcomes were measured by FFQ [32, 35–38, 42, 45], 24 h dietary recalls [32, 35–38, 45], food diary [38, 42], knowledge attitude practice (KAP) survey [32, 45], cognitive and attitudinal assessments [38], FV recall, number of days stayed, bought, ate healthy lunch at schools, snack scale [38], and 7-day recall [45], or 5-day food recall [42]. The intervention duration ranged from 15 min to 10 years [30, 36–38, 42, 45] and time of the follow up assessments ranged from immediately to four years after the intervention [30, 32, 36, 37, 42].

Four high-quality reviews (including unique 43 studies) reported “inconclusive” impacts on eating knowledge and behaviour, particularly in terms of healthy eating knowledge [44], intake of FV [34, 39, 40, 44], recommended fat [34, 39, 44], water [34, 40], processed snacks [40, 44], protein [40], fibre [44], and SSB [34]. These reviews exclusively focussed on interventions in HICs.

The **healthy eating education** components included lectures [34, 39, 40], group discussions [40, 44], games [39, 44], distribution of materials via compact discs (CDs), videos, emails, and text messages, blogs by a health coach [44], workshops for staff and students [39], and drama [40]. The contents involved healthy eating lessons [34, 39, 40, 44], food preparation, and taste testing [40].

The **school environment** components, working at the school system level, included canteen modifications [34, 39], food distributions [34], reduced price of fruits [44], social food marketing, staff trainings [39], loyalty programmes [34, 39], gardening, postering, and vending machine modifications [40]. The contents involved FV subscriptions [34, 40], increased availability of healthy food in canteen [34, 39], FV plantation [40], incentives to purchase healthy foods [34], staff training on healthy cooking healthy eating poster [39], healthy eating posters around schools [40], enhanced lunch sessions with healthy meals, and replacing SSBs with healthy foods [40].

The **family involvement** components included parents’ meetings and workshops [34, 39, 40], distribution of newsletters, CDs, magazines, calendars [39, 40, 44], engaging parents in school nutrition council groups [39], and loyalty programmes [39, 40]. The contents involved healthy eating lessons [34, 39, 40, 44], incentives to purchase healthy foods [34, 39], money rewards for purchasing healthy foods [40], cooking recipes [39], and healthy feeding to children [40].

The studies assessed in these four reviews employed RCTs (*n* = 39) [34, 39, 40, 44], non-RCTs (*n* = 16), cohort (*n* = 7), pre-post (*n* = 7) [34], and quasi-experimental (*n* = 2) designs [44]. The interventions were informed by TPB [34, 39, 40, 44], SCT [39, 40, 44], TTM [39, 44], ASE model, principles of interactive technology, SLT, health promotion model (Pender’s) [44], SEM, DIT, control theory, information-motivation-behavioural skills model [39], and community-based capacity building approach [40]. These outcomes were measured by 24-h recall [34, 39, 40, 44], FFQ [34, 40, 44], KAP [39], and 3-day food record [44]. The intervention duration ranged from 12 h to three years [34, 39, 40, 44] and only one review reported follow up assessments occurred ranged from immediately to three years after the intervention [34, 39, 40, 44].

Although none of the reviews mentioned that key stakeholders were involved in the intervention design process, only four reviews mentioned that engaging adolescents and key stakeholders in designing and implementing interventions is crucial to ensure their effectiveness [31, 33, 43, 44].

Overall, the combination of three BCTT hierarchical clusters was “likely effective”, as reported by two reviews with high methodological quality: shaping knowledge (workshops, games for students, homework for parents); associations (nutri-advice kiosks, entertainments such as drama, visits by inspiring personalities, SMS, emails, counselling via mHealth i.e., nutritional behavioural counselling); and antecedents (healthy eating club, school food marketing, canteen modification, such as increased availability of healthy foods, reduced fruit prices, parents’ meeting) (Supplementary file 5).

**Table 3:** Evidence on multi-component interventions

Author; year	Intervention design	Intervention description	Findings	Cochrane categorisation of effectiveness; JBI critical appraisal score
Bailey CJ et al., 2019 [36]	**Study design**: cross-sectional (*n* = 16), quasi- experimental (*n* = 13), qualitative (*n* = 7), mixed methods (*n* = 4), pre–post intervention (1), RCT (*n* = 1), longitudinal cohort (*n* = 1), observational (*n* = 1) **Theories**: SCT, TTM, TPB, HBM	**Healthy eating education components**: workshops, nutri-advice kiosk, cooking classes, quiz, games, field visits *Contents*: nutrition education, food safety, reading nutritional labels, healthy food purchase knowledge, farmers to visit schools to interact on healthy food cultivations **School environment change component**: school gardening, workshops with kitchen staff, canteen modification **Contents**: FV gardening, culinary lessons for kitchen staff and on-site chef consultations on healthy cooking, FV, milk, meat provision in canteen **Duration of interventions**: 1 week to 10 years **Follow-up range**: immediate to 2 years	97% of the included studies reported improved healthy eating knowledge, increased FV, decreased processed snacks intake	Promising; 8 (high quality)
Calvert S et al., 2019; [37]	**Study design**: RCT (*n* = 19), quasi-experimental (*n* = 7), cohort (*n* = 3) **Theories**: Not reported	**Healthy eating education components**: workshops, quiz, self-evaluation diary, self-assessment homework, entertainments, media shows (radio/TV), practical culinary lessons *Contents*: healthy eating education, handbooks, worksheets,, (e.g. problem solving, goal setting on healthy eating), computerised feedback, healthy cooking, media shows **School environment change components**: canteen modification *Contents*: increased availability of healthy foods **Family involvement components**: workshops, SMS, emails, homework, coupons *Contents*: information on healthy eating, heathy cooking via newsletters, feeding healthy foods to children, coupons for healthy food purchase **Duration of interventions**: 2 weeks to 3 years **Follow-up range**: 1 week to 4 years	83% of the included studies reported increased FV, decreased SSB, fat, and processed snack intake, improved intake of recommended calories and protein	Promising; 8 (high quality)
Champion KE et al., 2019 [44]	**Study design**: RCT (*n* = 14), quasi-experimental (*n* = 2) **Theories**: ASE model, Principles of interactive technology, SCT, SLT, TTM, TPB, HPM (pender’s),	**Healthy eating education components**:, online discussion boards, online games, SMS, emails, blog by health coach *Contents*: healthy eating lessons, knowledge and information via compact disc (CD), videos **School environment change component**: reduced price of fruit **Family involvement component**: healthy eating information handouts *Contents*: healthy eating information via newsletters, CD **Duration of interventions**: 1 month to 3 years **Follow-up range**: Immediately after intervention to 2 years	Inadequate evidence in improving healthy eating behaviour across all studies	No conclusion; 9 (high quality)
Hackman et al., 2014 [38]	**Study design**: RCT (*n* = 8), quasi-experimental (*n* = 2), pre-post (*n* = 1) **Theories**: TRA, TPB	**Healthy eating education components**: workshops, conference, campaign, games, quiz, SMS, entertainment, rewards *Contents*: healthy eating lessons, healthy cooking lesson, healthy menu planning, healthy foods as rewards, role play, creative writing on healthy eating, poster, comic workbooks, theatre play **School environment components**: school food marketing, food provision *Contents*: provision of FV, taste testing, healthy food promotion in school **Family involvement components**: healthy eating information handouts *Contents*: nutritional needs for adolescents via newsletters **Duration of interventions**: 15 min to 1 year **Follow-up range**: not reported	88% of included studies reported improved healthy eating knowledge and behaviour with increased FV, decreased snacks, high fat, SSB intake, increased intention for eating lunch in school	Promising; 8 (high quality)
McHugh C et al., 2020 [39]	**Study design**: RCT (*n* = 9) **Theories**: SCT, TTM, TPB, SEM, Diffusion of innovation theory, ASE model, control theory, IMBSM	**Healthy eating education components**: workshops for staff and students *Contents*: food and nutrition lessons, drama workshops on healthy eating **School environment change components**: canteen modification, social food marketing, staff training *Contents*: restriction of unhealthy foods, increased FV and healthy snacks, healthy food promotion, staff training on healthy cooking **Family involvement components**: events with parents (meetings, workshops, invite to school meals, including them in school nutrition council group), healthy eating information handouts, loyalty programs *Contents*: adolescents’ healthy eating, healthy cooking recipes, healthy eating information via calendars, newsletters, magazines, incentives to purchase healthy foods **Duration range**: 8 months to 3 years **Follow-up range**: 1–3 years	Inadequate evidence in improving healthy eating behaviour across all studies	No conclusion; 8 (high quality)
Medeiros et al., 2022 [45]	**Study design**: RCT (*n* = 24) **Theory**: Self-Regulation Theory, ASE Model, The action planning literature, Pedagogy of the Oppressed, by Paulo Freire, SCT, SEM, Bloom’s mastery of learning model, Bronfenbrenner’s ecological theory, TPB, TTM	**Healthy eating education components**: workshops *Contents*: healthy eating lessons **School environment change components**: canteen modification, postering, media marketing of healthy foods *Contents*: provision of healthy foods including FV, healthy eating posters around school premises, campaign on healthy eating **Family involvement components**: parents meeting, workshops, free healthy foods provision, *Contents*: healthy eating information discussion, leaflets, offering FV **Duration range**: 2 months to 3 years **Follow-up range**: Not reported	70% of the included reviews reported increased intake of FV, protein, healthy snacks	Promising; 11 (high quality)
Meiklejohn et al.; 2016 [40]	**Study design**: RCT (*n* = 13) **Theories**: SCT, TPB, Community-based capacity building approach	**Healthy eating education components**: workshops, games, entertainment *Contents*: healthy eating knowledge based lessons, food preparation, taste testing, drama **School environment change components**: gardening, postering, canteen modification, loyalty program *Contents*: FV gardening, posters display in lunch room on healthy eating, enhanced lunch session with healthy meals, replacing processed foods and SSBs with healthy foods and juice in vending machines, subscriptions (paying for regular access) to FV **Family involvement components**: parents’ meeting, loyalty program, healthy eating information handouts *Contents*: discussion on FV intake, meal preparation, money rewards for healthy feeding to their children, healthy eating information via newsletter, fact sheets, brochure, CD, magazine **Duration range**: 12 h to 12 weeks **Follow up range**: immediately after intervention to 2 years	Inadequate evidence in improving healthy eating behaviour across all studies	No conclusion; 9 (high quality)
Nakabayashi J et al., 2020 [42]	**Study design**: RCT (*n* = 8), quasi-experimental (*n* = 6) **Theory**: TTM	**Healthy eating education components**: workshops *Contents*: healthy eating knowledge, behaviour, and goal setting worksheets **Family involvement components**: healthy eating information handouts SMS *Contents*: healthy eating behaviour, nutritional guidelines for adolescents via magazines, letters **Duration range**: 1 h to 3 years **Follow up range**: 1 week to 2 years	86% of the included studies reported increased FV, decreased fat intake, balanced calorie intake	Promising; 7 (moderate quality)
Pierre CS et al., 2021 [43]	**Study design**: Not reported **Theories**: TTM, SCT, TPB, ASE model	**Healthy eating education components**: workshops, visits by inspiring personalities, games, SMS, healthy eating club *Contents*: healthy eating and nutrition lessons, cartoon-style nutrition handbook, visits by athletes, dancers, club activities (healthy cooking, drama, role-playing, poster making, photography exhibition on unhealthy eating) **School environment changes components**: school-wide food marketing *Contents*: SNaX messages- promotional displays via digital media, posters on healthy snacks **Family involvement components**: Parents meeting, homework *Contents*: healthy eating education for adolescents, feeding healthy foods to adolescents at home **Duration range**: 1 month-1 year **Follow-up range**: Not reported	All included studies reported improved healthy eating knowledge and behaviour including increased FV, decreased SSB intake, willingness to try new healthy foods, increased frequency of breakfast consumption	Likely effective; 9 (high quality)
Rose K et al., 2021 [31]	**Study desing**: Quasi-experimental (*n* = 11), RCT (*n* = 9), Qualitative (*n* = 4) mixed-method (*n* = 2), cross-sectional (*n* = 1) **Theories**: Not reported	**Healthy eating education component**: lectures, board game, instrumental SMS, nutri-active kiosks, drama, counselling via mHealth *Contents*: healthy eating, nutrition information via computer-generated tailored leaflet, nutritional behavioural counselling **School environment changes components: S**ocial food marketing, canteen modification *Contents*: daily free healthy meal, food choice towards plant based foods, chef demonstration, promotion of healthy snack purchases **Family involvement components**: Parents meeting *Contents*: healthy eating for adolescents **Duration of interventions**: Not reported **Follow-up range**: 4 weeks to 18 months	All included studies reported improved nutritional knowledge, increased FV, protein, decreased SSB, red meat, fat, processed snacks intake, improved frequency of breakfast consumption	Likely effective; 10 (high quality)
Sa JD & Lock K, 2008 [32]	**Study design**: RCT (*n* = 6), non-RCT (*n* = 1) **Theories**: Not reported	**Healthy eating education contents**: workshops, peer-leading activities *Contents*: lectures on healthy eating and its promotion, peer-leaders to promote healthy eating knowledge **School environment change components**: canteen modification, loyalty programmes, gardening *Contents*: increased provision of FV- free and/or subsidised, FV gardening **Family involvement components**: healthy eating information handouts *Contents*: healthy eating behaviour for adolescents via newsletters **Duration of interventions**: Not reported **Follow-up range**: 12 months to 3 years	70% of the included studies reported increased intake of FV	Promising; 9 (high quality)
Shinde et al., 2023 [30]	**Study design**: RCT (*n* = 19), CBA (*n* = 8) **Theories**: SCT, CBT, TPB, HBM, Pedagogy of the Oppressed, Health action process approach, HPS, TRA	**Healthy eating education components**: workshop, quiz, games, healthy eating information handouts,, culinary activities, entertainments *Contents*: healthy eating knowledge, role-plays, blackboard writing on healthy and unhealthy foods, food classifications, food label reading information via booklets, brochures, posters, magazines, webpage, puppet shows, movies, food tasting, healthy cooking recipe **School environment change components**: canteen modification, training for school staff *Contents*: daily sell of fresh fruits, nutrition training session **Family involvement components**: workshops, healthy eating information handouts information provision- *Contents*: healthy eating behaviour for adolescents via booklets, brochures, blackboard writings, posters, slogans, news leaflets, healthy recipe guides **Duration of interventions**: 7 days to 3 years **Follow-up range**: 8 weeks to 28 months	78% of the included studies reported improved healthy eating knowledge, increased FV, decreased SSB and processed food intake	Promising; 9 (high quality)
Van Cauwenberghe Evet al., 2010 [34]	**Study design**: RCT (*n* = 5), non-RCT (*n* = 5), prospective cohort (*n* = 2), pre-post (*n* = 1) **Theories**: TPB	**Healthy eating education components**: workshops *Contents*: healthy eating lessons **School environment change components**: canteen modification,, loyalty programs *Contents*: healthy foods in canteen, FV distributions, subscription (paying for regular access) to healthy foods, and incentives for purchasing healthy foods **Family involvement components**: Parents meeting *Content*: discussion on promoting healthy eating behaviour among their children **Duration of interventions**: 1 week to 2 years **Follow-up range**: 2 weeks to 2 years	Inadequate evidence in improving healthy eating behaviour across all studies	No conclusion; 8 (high quality)
Vézina-Im LA et al., 2017 [35]	**Study design**: RCT (*n* = 13), quasi-experimental (*n* = 11), pre–post (*n* = 12) Theories: SCT, TPB, DIT, ET, SDT, ELM, SRT, TIT	**Curriculum components**: workshops *Contents*: consequences of SSB intake, healthy eating goal setting, self-monitoring of eating behaviour **School environment change components**: canteen modification *Contents*: replacing SSB with healthier alternatives (milk, juice, water) in vending machine **Family and community involvement component**: parents’ meetings, social support groups, healthy eating information handouts *Contents*: healthy eating knowledge, parents and family involvement to share experience, challenges and encourage healthy eating behaviour, information distribution via newsletter, emails, postcards **Duration range**: not reported **Follow-up range**: not reported	72% of the included studies reported decreased intake of SSB	Promising; 9 (high quality)

RCT: Randomised Controlled Trial; CBA: Controlled before-after; FV: Fruit and Vegetable; ASE: Attitude, social influence and self-efficacy Model; SCT: Social Cognitive Theory; TTM: Trans-theoretical Model; TPB: Theory of Planned Behaviour; HBM: Health Belief Model; HPM: Health Promotion Model; SLT: Social Learning Theory; SSB: Sugar-sweetened Beverage; SEM: Socio-ecological Model; IMBSM: Information-Motivation Behavioural Skills Model; CBT: Cognitive Behavioural Theory; HPS: Health Promoting School; DIT: Diffusion of Innovations Theory; ET: Expectancy Theory; SDT: Self-determination Theory; ELM: Elaboration Likelihood Model; SRT: Self-regulation Theory; TIT: Theory of Interpersonal Behaviour; SMS: short message service

## Discussion

To our knowledge, this umbrella review represents the first comprehensive synthesis of evidence on the effectiveness of school-based healthy eating interventions targeting adolescents aged 10 to 19 years. Most (83%) of the reviews were of high methodological quality, providing confidence in the findings. The majority (71%) of high-quality reviews assessing multi-component interventions reported “promising” to “likely effectiveness”, suggesting that a combination of individual- and system-level interventions is most effective in promoting healthy eating among adolescents. This finding is consistent with recent empirical evidence [46–49], highlighting the potential of such interventions to address the complex factors influencing adolescents’ eating behaviour. Our review found that curricula driven by technology effectively encouraged healthy eating behaviours at the individual level, a finding further reinforced by recent studies [50–52]. However, our review also found that the broader impact and sustainability of individual-level interventions are contingent upon their integration into the system-level interventions that include changing the school environment to improve availability of healthy foods and involving families. Combining system and individual-level interventions can create supportive environments that underpin and perpetuate changes in individual behaviour [17, 19, 53, 54]. Reviews examining school-based healthy eating interventions for a wider age range, including both children and adolescents, reveal different emphases. Effective intervention components focusing on children emphasise antecedents, particularly parental involvement in shaping eating behaviour and the importance of healthy food accessibility [32, 34, 49]. However, our review found that the combination of effective components within the collaborative individual- and system-level approach for adolescents aged 10 to 19 years involved shaping knowledge through educational instructions and experiments, creating associations with stimuli that cue healthy behaviours, and establishing antecedents to facilitate healthy food choices [17, 19, 53, 54].

The evidence synthesised in this review was primarily from studies in HICs (87%) that did not differentiate between geographical contexts. As interventions are likely to be context specific, this limits its applicability to LMICs. For example, the socioeconomic and infrastructural differences between HICs and LMICs may influence the effectiveness and feasibility of interventions [54–56] or limited access to technology, financial resources, and trained personnel in LMICs may hinder the successful implementation of tech-driven as well as multi-component interventions that have been promising in HICs [29, 43, 54–56]. However, the evidence from HICs in this umbrella review still provides valuable insights and a foundation for future research and intervention development in resource-limited settings. The components and contents of effective interventions identified in HICs, such as the importance of multi-component approaches with the potential of technology-based strategies, are a starting point for designing and testing school-based healthy eating interventions in LMICs. However, these interventions will require adaptation and contextualisation to the constraints and opportunities in LMICs.

The limited number of reviews reporting on stakeholder involvement in intervention development underscores a critical gap in the current literature. Empirical evidence suggests that engaging key stakeholders, including adolescents, parents, teachers, and policy experts, in intervention design ensures tailoring to adolescents’ needs, feasibility, and successful implementation [57–59]. Adolescents offer insights into their eating habits and preferences [57–59], parents shape their children’s eating behaviours [59, 60], teachers ensure compatibility with school resources [59], and policymakers promote policies for long-term support [59, 61].

The scarcity of reviews reporting on the feasibility of these intervention exposes a significant gap in the current literature. Although a few reviews suggest that technology-driven interventions may be feasible [29, 41], recent studies have identified several challenges that undermine their feasibility. These challenges include teachers’ lack of understanding of the operating systems of the technology, limited internet access, and poor technology infrastructure [51, 52]. Furthermore, the feasibility of multi-component interventions that require additional resources has been questioned [52, 62], which is consistent with the reporting from one review [39]. This highlights the need for more comprehensive feasibility assessments to identify and address the logistical, contextual, and stakeholder factors that influence intervention effectiveness [62, 63].

Inconsistent reporting across reviews made it difficult to determine if effectiveness varied based on theoretical underpinnings. While psychosocial theories, such as SCT, SLT, and TPB were most commonly used to inform the interventions, these interventions did not incorporate behaviour change taxonomy technique (BCTT) [64, 65]. Literature suggests using BCTT with behaviour change theories and frameworks, such as goal setting theory, TTM, TRA, and Capability, Opportunity, Motivation-Behaviour (COM-B) framework, for more effective and sustainable behaviour change [64–67]. BCTT can improve intervention designs, enable cross-study analysis, and inform implementation feasibility [64, 68, 69].

The interventions assessed in the reviews relied on survey methods, mostly FFQs and food recalls. These outcome measures are prone to biases and inaccuracies, due to recall bias, social desirability bias (i.e., providing answers they perceive as more socially acceptable rather than accurate), short-term dietary variability, challenges in estimating portion sizes, limited food options, and seasonal variation [70]. Triangulating this data with data from wearable tech, mobile apps, and school canteen sales data, ecological momentary assessment (EMA) for real-time data collection, direct observation of adolescents’ eating behaviours, and proxy reports from family members can provide insights into behaviour changes [70–76]. However, the acceptability and feasibility of some of methods should be explored within the context of resource availability prior to implementation.

This umbrella review stands out for two key strengths. First, it provides a comprehensive narrative synthesis of the evidence while critically examining methodological gaps in intervention designs. Second, it goes beyond a simple narrative compilation of findings by employing the HPS framework, TIDieR framework, and an intervention effectiveness categorisation system to synthesise intervention components, contents, and their effectiveness. However, our review had some limitations. The included reviews had mixed methodological quality, and many included low-quality primary studies. Our findings may also be influenced by the heterogeneity of the intervention designs of selected reviews and inconsistent reporting of intervention characteristics. Our umbrella review included English-language peer-reviewed reviews, excluding literature in other languages and grey literature. Therefore, we might have missed reviews published in other languages or as grey literature. This may have also led to an overestimation of the interventions’ effectiveness due to publication bias [77, 78].

## Conclusion

Multi-component school-based healthy eating interventions have shown promising results in improving healthy eating knowledge and behaviour among adolescents aged 10 to 19 years, particularly when combining individual- and systemic-level approaches. However, this umbrella review highlighted a significant gap in evidence from LMICs and a lack of participatory approach in designing and implementing the interventions. The limited and inconsistent reporting on intervention characteristics and strategies emphasises the need for comprehensive and high-quality systematic reviews of primary studies. Such reviews would allow for the consolidation of evidence from all types of school-based healthy eating interventions and the investigation of specific intervention components’ effectiveness. Addressing these gaps is crucial for developing effective and sustainable interventions to promote healthy eating among adolescents worldwide.

## Electronic supplementary material

Below is the link to the electronic supplementary material.

Supplementary Material 1

Supplementary Material 2

Supplementary Material 3

Supplementary Material 4

Supplementary Material 5

## Data Availability

Not applicable.
