# Supplementary material for: School-based healthy eating interventions for adolescents aged 10–19 years: an umbrella review
Source: Int J Behav Nutr Phys Act. 2024 Oct 14;21:117. doi: 10.1186/s12966-024-01668-6 (PMC11472496; doi:10.1186/s12966-024-01668-6)
Supplement: Supplementary file 2 — Supplementary Material 2 [file 12966_2024_1668_MOESM2_ESM.docx]

**Search details**

All databases searched on 30^th^ December 2023

**PubMed search strategy: 4324**

1#  (((((School health service[MeSH terms]) OR School [MeSH Terms]) OR Teacher-led OR Peer-led)

2#  (((((((Adolescents[MeSH Terms]) OR Children[MeSH Terms]) OR Student[MeSH Terms]) OR teenage [MeSH Terms]) OR Young adult[MeSH Terms]) OR young people OR Youth [MeSH Terms]))

3#  ((((((((((((((((healthy diet[MeSH Terms]) OR nutrition[MeSH Terms]) OR fruit[MeSH Terms]) OR vegetable[MeSH Terms]) OR sugar[MeSH Terms]) OR calorie) OR protein[MeSH Terms]) OR milk[MeSH Terms]) OR dairy) OR dietary behaviour) OR diet pattern) OR healthy eating[MeSH Terms]) OR diet[MeSH Terms]) OR food[MeSH Terms]) OR snacks[MeSH Terms]) OR processed) OR beverage[MeSH Terms]

4# (((((Review) OR Research synthesis) OR Meta-analysis) OR Meta synthesis))

5# (1# AND 2# AND 3# AND 4#)

**Science direct search strategy: 406**

(“School health service”) AND (“Adolescents” OR “children” OR “students” OR) AND (“healthy diet” OR “nutrition” OR “dietary behavior”) AND (“review” OR “Meta-analysis”)

**CINAHL search strategy: 14**

S1 TI School

S2 TI school health service

S3 TI Teacher-led

S4 TI Peer-led

S5 MW Adolescent

S6 MW children

S7 MW Student

S8 MH Young adult

S9 MH adult

S10 TI Young people

S11 TI youth

S12 MH Nutrition

S13 TI calorie

S14 MW protein

S15 MH milk

S16 MW dairy

S17 MW snacks

S18 TI processed

S19 TI beverage

S20 TI Healthy diet

S21 MH Fruit

S22 MW vegetable

S23 MW Sugar

S24 TI Dietary behavior

S25 TI Diet pattern

S26 TI Healthy eating

S27 MH Diet

S28 MH Food

S29 TI Review

S30 TI Research synthesis

S31 MW Meta-analysis

S32 TI Meta synthesis

S33 (S1 OR S2 OR S3 OR S4)

S34 (S5 OR S6 OR S7 OR S8 OR S9 OR S10 OR S11)

S35 (S12 OR S13 OR S14 OR S15 OR S16 OR S17 OR S18 OR S19 OR S20 OR S21 OR S22 OR S23 OR S24 OR S25 OR S26 OR S27 OR S28)

S36 (S29 OR S30 OR S31 OR S32)

S37 (S33 AND S34 AND S35 AND S36)

**Cochrane systematic review search strategy: 5**

#1 MeSH descriptor: [School Health Services] this term only

#2 MeSH descriptor: [School] this term only

#3 (Teacher-led):ti,ab,kw

#4 (peer-led):ti,ab,kw

#5 MeSH descriptor: [Adolescent] this term only

#6 MeSH descriptor: [Children] this term only

#7 MeSH descriptor: [Students] this term only

#8 (teenage) :ti,ab,kw

#9 (Young Adult) :ti,ab,kw

#10 (Young people) :ti,ab,kw

#11 (Youth) :ti,ab,kw

#12 MeSH descriptor: [Adult] this term only

#13 MeSH descriptor: [Diet, Healthy] this term only

#14 MeSH descriptor: [Fruit] this term only

#15 MeSH descriptor: [Vegetable] this term only

#16 MeSH descriptor: [Sugars] this term only

# 17 (calorie):ti,ab,kw

# 18 MeSH descriptor: [Proteins] this term only

# 19 MeSH descriptor: [Milk] this term only

# 20 MeSH descriptor: [Dairy product] this term only

# 21 (dietary behaviour):ti,ab,kw

# 22 (diet pattern):ti,ab,kw

# 23 MeSH descriptor: [Snacks] this term only

# 24 (processed):ti,ab,kw

# 25 MeSH descriptor: [Beverages] this term only

#26 MeSH descriptor: [Diet] this term only

#27 MeSH descriptor: [Food] this term only

#28 (Review):ti,ab,kw

#29 (Research synthesis):ti,ab,kw

#30 (Meta-analysis):ti,ab,kw

#31 (Meta synthesis):ti,ab,kw

#32 (#1 OR #2 OR #3 OR #4)

#33 (#5 OR #6 OR #7 OR #8 OR #9 OR #10 OR #11 OR #12)

#34 (#13 OR #14 OR #15 OR #16 OR #17 OR #18 OR #19 OR #20 OR #21 OR #22 OR #23 OR #24 OR #25 OR #26 OR #27)

#35 (#28 OR #29 OR #30 OR #31)

#36 (#32 AND #33 AND #34 AND #35)

**Scopus search strategy: 6928**

TITLE-ABS-KEY(("School health service" OR "school" OR "Peer-led" OR "Teacher-led" ) AND ( "Adolescent" OR "Children" OR "Student" OR "young-adult" OR "teenage" OR "youth" OR "Young people" ) AND ( "Healthy diet" OR "nutrition" OR "Fruit" OR "Vegetable" OR "sugar" OR "calorie" OR "protein" OR "milk" OR "dairy" OR "dietary behaviour" OR "diet pattern" OR "healthy eating" OR "diet" OR "Food" OR "snacks" OR "processed" OR "beverage" ) AND ( "Review" OR "Meta-analysis" OR "research synthesis" OR "Meta synthesis" ))

**Eric search strategy: 269**

(“School health service” OR “school” OR “Peer-led” OR “Teacher-led”) AND (“Adolescent” OR “Children” OR “Student” OR “young-adult” OR “teenage” OR “youth” OR “Young people”) AND (“Healthy diet” OR “nutrition” OR “Fruit” OR “Vegetable” OR “sugar” OR “calorie” OR “protein” OR “milk” OR “dairy” OR “dietary behaviour” OR “diet pattern” OR “healthy eating” OR “diet” OR “Food” OR “snacks” OR “processed” OR “beverage”) AND (“Review” OR “Meta-analysis” OR “research synthesis” OR “Meta synthesis”)

**Web of Science: 2405**

( "School health service" OR "school" OR "Peer-led" OR "Teacher-led" ) AND ( "Adolescent" OR "Children" OR "Student" OR "young-adult" OR "teenage" OR "youth" OR "Young people" ) AND ( "Healthy diet" OR "nutrition" OR "Fruit" OR "Vegetable" OR "sugar" OR "calorie" OR "protein" OR "milk" OR "dairy" OR "dietary behaviour" OR "diet pattern" OR "healthy eating" OR "diet" OR "Food" OR "snacks" OR "processed" OR "beverage" ) AND ( "Review" OR "Meta-analysis" OR "research synthesis" OR "Meta synthesis")

**JBI Database of Systematic Reviews and Implementation Reports: 128**

( "School health service" OR "school" OR "Peer led" OR "Teacher led" ) AND ( "Adolescent" OR "Children" OR "Student" OR "young adult" OR "teenage" OR "youth" OR "Young people" ) AND ( "Healthy diet" OR "nutrition" OR "Fruit" OR "Vegetable" OR "sugar" OR "calorie" OR "protein" OR "milk" OR "dairy" OR "dietary behaviour" OR "diet pattern" OR "healthy eating" OR "diet" OR "Food" OR "snacks" OR "processed" OR "beverage" ) AND ( "Review" OR "Meta analysis" OR "research synthesis" OR "Meta synthesis")

**Ovid (Embase, Medline, PsycINFO) search strategy: (Keyword)**

**Ovid (Embase): 4264**

1 exp school health service/

2 exp school/

3 exp controlled study/ or exp teaching/ or exp teacher/ or exp peer group/

4 exp student/ or exp high school student/ or exp health student/ or exp middle school student/

5 exp adolescent/

6 exp young adult/

7 exp child/

8 exp feeding behavior/ or exp eating/ or exp health promotion/

9 exp nutrition education/ or exp child nutrition/ or exp nutrition service/ or exp adolescent nutrition/ or exp nutrition/ or exp nutrition policy/

10 exp fruit/ or exp fruit vegetable/ or exp "fruit and vegetable juice"/ or exp fruit consumption/

11 exp vegetable/ or exp vegetable juice/ or exp vegetable consumption/

12 exp sugar intake/ or exp sugar/ or exp sugar-sweetened beverage/

13 exp high calorie diet/ or exp calorie/ or exp low calorie diet/

14 exp protein intake/ or exp protein/

15 exp milk/

16 exp dairy product/

17 exp dietary pattern/

18 1exp diet/ or exp healthy diet/

19 exp food composition/ or exp food/ or exp food quantity/ or exp health food/ or exp food addiction/ or exp junk food/ or exp food processing/ or exp processed food/ or exp food intake/ or exp instant food/ or exp food quality/ or exp fast food/

20 exp sweetened beverage/ or exp beverage/ or exp artificially sweetened beverage/ or exp sugar-sweetened beverage/ or exp carbonated beverage/

21 exp systematic review/ or exp review/

22 exp qualitative research/ or exp synthesis/

23 exp meta analysis/

24 1 or 2 or 3

25 4 or 5 or 6 or 7

26 8 or 9 or 10 or 11 or 12 or 13 or 14 or 15 or 16 or 17 or 18 or 19 or 20

27 21 or 22 or 23

28 24 and 25 and 26 and 27

**Ovid (PsycINFO): 688**

1 exp School Nurses/ or exp Schools/ or exp School Based Intervention/ or exp Health Education/ or exp Health Promotion/ or exp Adolescent Health/

2 exp Junior High Schools/ or exp High Schools/ or exp Middle Schools/ or exp Schools/ or exp Elementary Schools/

3 exp Computer Assisted Instruction/ or exp Classrooms/ or exp Teachers/ or exp Elementary School Students/ or exp Elementary Schools/ or exp School Based Intervention/ or exp Teaching/

4 exp Health Promotion/ or exp Program Evaluation/ or exp Peers/ or exp Academic Achievement/ or exp Peer Counseling/ or exp Health Education/ or exp Educational Programs/

5 exp Adolescent Behavior/ or exp Adolescent Attitudes/ or exp Adolescent Health/
6 exp Child Care/ or exp Child Behavior/ or exp Child Health/
7 exp Only Children/

8 exp Junior High School Students/ or exp High School Students/ or exp Kindergarten Students/ or exp Middle School Students/ or exp Primary School Students/ or exp Intermediate School Students/ or exp Students/ or exp Nursery School Students/ or exp Elementary School Students/

9 exp Eating Behavior/ or exp Food/ or exp Health Behavior/ or exp Food Intake/ or exp Nutrition/ or exp Diets/ or exp Health Promotion/

10 exp Calories/

11 exp Protein/

12 1exp Food Preferences/ or exp Eating Attitudes/ or exp Food Addiction/

13 exp Sugars/
14 exp "Beverages (Nonalcoholic)"/

15 exp "Literature Review"/ or exp "Systematic Review"/

16 exp Meta Analysis/ or exp Intervention/

17 1 or 2 or 3 or 4

18 5 or 6 or 7 or 8

19 9 or 10 or 11 or 12 or 13 or 14

20 15 or 16

21 17 and 18 and 19 and 20

**Ovid (Medline): 350**

1 exp School Health Services/

2 exp Schools/

3 exp Peer Group/

4 exp Adolescent Behavior/ or exp "National Longitudinal Study of Adolescent Health"/ or exp Adolescent/ or exp Adolescent Health Services/ or exp Adolescent Nutritional Physiological Phenomena/ or exp Adolescent Health/

5 exp Child Health/ or exp Child Nutritional Physiological Phenomena/ or exp Child/ or exp Child Behavior/ or exp Child Health Services/ or exp Child Nutrition Disorders/

6 exp Students/

7 exp Young Adult/

8 exp Diet, Healthy/

9 exp Nutrition Policy/ or exp Nutrition Assessment/ or exp Nutririon

10 exp "Fruit and Vegetable Juices"/ or exp Fruit/

11 exp Vegetables/
12 exp Sugars/

13 exp Energy Intake/

14 exp Proteins/

15 exp Milk/

16 exp Dairy Products/

17 exp Diet/ or exp Feeding Behavior/

18 exp Food/ or exp Food Addiction/

19 exp Snacks/

20 exp Beverages/

21 exp "Systematic Review"/ or exp "Review"/

22 exp Meta-Analysis/

23 1 or 2 or 3

24 4 or 5 or 6 or 7

25 8 or 9 or 10 or 11 or 12 or 13 or 14 or 15 or 16 or 17 or 18 or 19 or 20

26 21 or 22
27 23 and 24 and 25 and 26
