# Supplementary material for: School-based healthy eating interventions for adolescents aged 10–19 years: an umbrella review
Source: Int J Behav Nutr Phys Act. 2024 Oct 14;21:117. doi: 10.1186/s12966-024-01668-6 (PMC11472496; doi:10.1186/s12966-024-01668-6)
Supplement: Supplementary file 3 — Supplementary Material 3 [file 12966_2024_1668_MOESM3_ESM.docx]

**Supplementary file C**

Table: Citation Matrix

|  | **No of overlapping articles** | **Calvert, 2019** | **Van Cauwenberghe, 2010** | **Meiklejohn, 2016** | **Alcantara, 2018** | **Champion, 2019** | **Vezina-lm, 2017** | **Bailey, 2019** | **Tallon, 2019** | **McHugh, 2020** | **Rose, 2020** | **Nakabayashi, 2020** | **Sa JD, 2008** | **Melo GRDA, 2017** | **Medeiros 2022** | **Hackman,** | **Pierre** | **Shinde, 2023** |
| --- | --- | --- | --- | --- | --- | --- | --- | --- | --- | --- | --- | --- | --- | --- | --- | --- | --- | --- |
| **Unique primary studies** |  | **29** | **13** | **11** | **8** | **11** | **36** | **44** | **13** | **4** | **27** | **14** | **7** | **11** | **24** | **11** | **53** |  |
| Aceves‐Martins 2017 | 2 | x |  |  |  |  |  |  |  |  | x |  |  |  |  |  |  |  |
| Bere E 2006 | 3 | x |  | x |  |  |  |  |  |  |  |  | x |  |  |  |  |  |
| Birnbaum 2002 | 3 | x |  | x |  |  |  |  |  |  |  |  |  |  | x |  |  |  |
| Bukhari 2011 | 3 | x |  |  |  |  |  |  |  |  |  |  |  |  | x |  | x |  |
| Chin 2008 |  | x |  |  |  |  |  |  |  |  |  |  |  |  |  |  |  |  |
| de Visser 2016 |  | x |  |  |  |  |  |  |  |  |  |  |  |  |  |  |  |  |
| Dowd 2015 |  | x |  |  |  |  |  |  |  |  |  |  |  |  |  |  |  |  |
| Dzewaltowski 2009 | 2 | x |  |  |  |  |  |  |  |  |  |  |  |  | x |  |  |  |
| Foley BC 2017 | 2 | x |  |  |  |  |  |  |  |  |  |  |  |  |  |  | x |  |
| Gratton L 2007 | 4 | x | x |  |  |  |  |  |  |  |  |  |  |  | x | x |  |  |
| Haerens 2006 | 4 | x |  | x |  | x | x |  |  |  |  |  |  |  |  |  |  |  |
| Hölund 1990 |  | x |  |  |  |  |  |  |  |  |  |  |  |  |  |  |  |  |
| Hoelscher 2016 |  | x |  |  |  |  |  |  |  |  |  |  |  |  |  |  |  |  |
| Lien 2010 |  | x |  |  |  |  |  |  |  |  |  |  |  |  |  |  |  |  |
| Lo E 2008 | 3 | x |  |  |  |  | x |  |  |  |  |  |  |  |  |  | x |  |
| Lubans 2009 |  | x |  |  |  |  |  |  |  |  |  |  |  |  |  |  |  |  |
| Maatoug 2015 |  | x |  |  |  |  |  |  |  |  |  |  |  |  |  |  |  |  |
| Martens 2010 |  | x |  |  |  |  |  |  |  |  |  |  |  |  |  |  |  |  |
| Mauriello 2010 | 3 | x |  |  |  |  |  |  | x |  |  | x |  |  |  |  |  |  |
| McCabe 2015 |  | x |  |  |  |  |  |  |  |  |  |  |  |  |  |  |  |  |
| Neumark‐Sztainer 1995 |  | x |  |  |  |  |  |  |  |  |  |  |  |  |  |  |  |  |
| Perry 1987 |  | x |  |  |  |  |  |  |  |  |  |  |  |  |  |  |  |  |
| Ratcliffe 2011 | 2 | x |  |  |  |  |  | x |  |  |  |  |  |  |  |  |  |  |
| Revill 2004 |  | x |  |  |  |  |  |  |  |  |  |  |  |  |  |  |  |  |
| Siega‐Riz 2011 |  | x |  |  |  |  |  |  |  |  |  |  |  |  |  |  |  |  |
| Tsorbatzoudis 2005 | 3 | x | x |  |  |  |  |  |  |  |  |  |  |  |  | x |  |  |
| Wang 2015 | 3 | x |  |  |  |  |  |  |  |  |  |  |  |  | x |  |  | x |
| Wilson 2012 |  | x |  |  |  |  |  |  |  |  |  |  |  |  |  |  |  |  |
| Yang 2015 | 3 | x |  |  |  |  |  |  | x |  |  |  |  | x |  |  |  |  |
| Haerens 2007 | 6 |  | x | x |  |  |  |  | x |  |  | x | x | x | x |  |  |  |
| Hassapidou 1997 | 2 |  | x |  |  |  |  |  |  |  |  |  |  |  | x |  |  |  |
| Klepp 1993 | 2 |  | x |  |  |  |  | x |  |  |  |  |  |  |  |  |  |  |
| Martens 2005 |  |  | x |  |  |  |  |  |  |  |  |  |  |  |  |  |  |  |
| Martens 2006 |  |  | x |  |  |  |  |  |  |  |  |  |  |  |  |  |  |  |
| Martens 2008 | 2 |  | x |  |  |  |  |  |  |  |  |  |  |  | x |  |  |  |
| Eicchorn 2007 |  |  | x |  |  |  |  |  |  |  |  |  |  |  |  |  |  |  |
| Passmore 2005 |  |  | x |  |  |  |  |  |  |  |  |  |  |  |  |  |  |  |
| Ask 2006 |  |  | x |  |  |  |  |  |  |  |  |  |  |  |  |  |  |  |
| Loughridge 2005 |  |  | x |  |  |  |  |  |  |  |  |  |  |  |  |  |  |  |
| Parker |  |  | x |  |  |  |  |  |  |  |  |  |  |  |  |  |  |  |
| Prell 2005 | 2 |  | x | x |  |  |  |  |  |  |  |  |  |  |  |  |  |  |
| Young |  |  | x |  |  |  |  |  |  |  |  |  |  |  |  |  |  |  |
| Bjelland 2011 |  |  |  | x |  |  |  |  |  |  |  |  |  |  |  |  |  |  |
| Dewar 2013 |  |  |  | x |  |  |  |  |  |  |  |  |  |  |  |  |  |  |
| Foster 2008 | 2 |  |  | x |  |  |  |  |  | x |  |  |  |  |  |  |  |  |
| Hoppu 2010 | 4 |  |  | x |  |  |  |  |  | x | x |  |  |  | x |  |  |  |
| Lytle 2004 | 4 |  |  | x |  |  |  |  |  | x |  |  | x |  | x |  |  |  |
| Mihas 2009 | 4 |  |  | x |  |  |  |  |  |  | x |  |  |  | x |  | x |  |
| Millar 2011 |  |  |  | x |  |  |  |  |  |  |  |  |  |  |  |  |  |  |
| Te Velde 2008 |  |  |  | x |  |  |  |  |  |  |  |  |  |  |  |  |  |  |
| Dunn 2004 |  |  |  |  | x |  |  |  |  |  |  |  |  |  |  |  |  |  |
| Long 2004 | 3 |  |  |  | x |  |  | x | x |  |  |  |  |  |  |  |  |  |
| Whittemore 2013 | 4 |  |  |  | x | x | x |  |  |  |  |  |  | x |  |  |  |  |
| Whittemore 2013 (different study) |  |  |  |  | x |  |  |  |  |  |  |  |  |  |  |  |  |  |
| Cullen 2013 |  |  |  |  | x |  |  |  |  |  |  |  |  |  |  |  |  |  |
| Majumdar 2013 |  |  |  |  | x |  |  |  |  |  |  |  |  |  |  |  |  |  |
| Fraticelli 2016 |  |  |  |  | x |  |  |  |  |  |  |  |  |  |  |  |  |  |
| Turnin 2016 | 3 |  |  |  | x |  |  | x |  |  | x |  |  |  |  |  |  |  |
| Brick 2015. |  |  |  |  |  | x |  |  |  |  |  |  |  |  |  |  |  |  |
| Brick 2017 | 2 |  |  |  |  | x |  |  |  |  |  | x |  |  |  |  |  |  |
| Velicer 2013 |  |  |  |  |  | x |  |  |  |  |  |  |  |  |  |  |  |  |
| Casazza 2007 | 2 |  |  |  |  | x |  |  | x |  |  |  |  |  |  |  |  |  |
| Ezendam 2012 | 2 |  |  |  |  | x |  |  |  |  |  |  |  | x |  |  |  |  |
| Frenn 2003 |  |  |  |  |  | x |  |  |  |  |  |  |  |  |  |  |  |  |
| Frenn 2005 | 2 |  |  |  |  | x |  |  | x |  |  |  |  |  |  |  |  |  |
| Lana 2014 |  |  |  |  |  | x |  |  |  |  |  |  |  |  |  |  |  |  |
| Mauriello 2006 |  |  |  |  |  | x |  |  |  |  |  |  |  |  |  |  |  |  |
| Muzaffar 2019 | 2 |  |  |  |  | x |  |  |  |  |  | x |  |  |  |  |  |  |
| Prochaska 2004 |  |  |  |  |  | x |  |  |  |  |  |  |  |  |  |  |  |  |
| Bae 2012 |  |  |  |  |  |  | x |  |  |  |  |  |  |  |  |  |  |  |
| Bauhoff 2014 |  |  |  |  |  |  | x |  |  |  |  |  |  |  |  |  |  |  |
| Blum 2008 |  |  |  |  |  |  | x |  |  |  |  |  |  |  |  |  |  |  |
| Bogart 2011 | 2 |  |  |  |  |  | x |  |  |  |  |  |  |  |  |  | x |  |
| Casazza 2006 |  |  |  |  |  |  | x |  |  |  |  |  |  |  |  |  |  |  |
| Collins 2014 |  |  |  |  |  |  | x |  |  |  |  |  |  |  |  |  |  |  |
| Contento 2010 | 2 |  |  |  |  |  | x |  |  |  |  |  |  |  |  |  | x |  |
| Cordeira 2012 |  |  |  |  |  |  | x |  |  |  |  |  |  |  |  |  |  |  |
| Cradock 2011 |  |  |  |  |  |  | x |  |  |  |  |  |  |  |  |  |  |  |
| Cullen 2008 |  |  |  |  |  |  | x |  |  |  |  |  |  |  |  |  |  |  |
| da Silva 2011 |  |  |  |  |  |  | x |  |  |  |  |  |  |  |  |  |  |  |
| Davis 2007 |  |  |  |  |  |  | x |  |  |  |  |  |  |  |  |  |  |  |
| Dubuy 2014 | 2 |  |  |  |  |  | x |  |  |  |  |  |  |  |  |  | x |  |
| Greece 2011 |  |  |  |  |  |  | x |  |  |  |  |  |  |  |  |  |  |  |
| Jones 2014 |  |  |  |  |  |  | x |  |  |  |  |  |  |  |  |  |  |  |
| Lao LS 2011 |  |  |  |  |  |  | x |  |  |  |  |  |  |  |  |  |  |  |
| Malbon 2012 |  |  |  |  |  |  | x |  |  |  |  |  |  |  |  |  |  |  |
| McGoldrick 2006 |  |  |  |  |  |  | x |  |  |  |  |  |  |  |  |  |  |  |
| Nanney 2014 |  |  |  |  |  |  | x |  |  |  |  |  |  |  |  |  |  |  |
| Nanney 2016 |  |  |  |  |  |  | x |  |  |  |  |  |  |  |  |  |  |  |
| Neumark-Sztainer 2010 |  |  |  |  |  |  | x |  |  |  |  |  |  |  |  |  |  |  |
| Patel 2011 |  |  |  |  |  |  | x |  |  |  |  |  |  |  |  |  |  |  |
| Pbert 2013 |  |  |  |  |  |  | x |  |  |  |  |  |  |  |  |  |  |  |
| Singhal 2010 |  |  |  |  |  |  | x |  |  |  |  |  |  |  |  |  |  |  |
| Smith 2014 |  |  |  |  |  |  | x |  |  |  |  |  |  |  |  |  |  |  |
| Teufel 1998 |  |  |  |  |  |  | x |  |  |  |  |  |  |  |  |  |  |  |
| Thiele 1989 |  |  |  |  |  |  | x |  |  |  |  |  |  |  |  |  |  |  |
| Winett 1999 |  |  |  |  |  |  | x |  |  |  |  |  |  |  |  |  |  |  |
| Wing 2015 |  |  |  |  |  |  | x |  |  |  |  |  |  |  |  |  |  |  |
| Woodward-Lopez 2010 |  |  |  |  |  |  | x |  |  |  |  |  |  |  |  |  |  |  |
| Wordell 2012 |  |  |  |  |  |  | x |  |  |  |  |  |  |  |  |  |  |  |
| Yildirim 2013 |  |  |  |  |  |  | x |  |  |  |  |  |  |  |  |  |  |  |
| Dewhurst 2011 |  |  |  |  |  |  |  | x |  |  |  |  |  |  |  |  |  |  |
| Dewhurst 2008 |  |  |  |  |  |  |  | x |  |  |  |  |  |  |  |  |  |  |
| Gracey 1996 |  |  |  |  |  |  |  | x |  |  |  |  |  |  |  |  |  |  |
| Jaenke 2012 |  |  |  |  |  |  |  | x |  |  |  |  |  |  |  |  |  |  |
| Morgan 2010 |  |  |  |  |  |  |  | x |  |  |  |  |  |  |  |  |  |  |
| Pendergast 2012 |  |  |  |  |  |  |  | x |  |  |  |  |  |  |  |  |  |  |
| Ronto 2016 |  |  |  |  |  |  |  | x |  |  |  |  |  |  |  |  |  |  |
| Ronto 2016 (different study) |  |  |  |  |  |  |  | x |  |  |  |  |  |  |  |  |  |  |
| Ronto 2016 (different study) |  |  |  |  |  |  |  | x |  |  |  |  |  |  |  |  |  |  |
| Slater 2013 |  |  |  |  |  |  |  | x |  |  |  |  |  |  |  |  |  |  |
| Zhou 2016 |  |  |  |  |  |  |  | x |  |  |  |  |  |  |  |  |  |  |
| Petralias 2016 |  |  |  |  |  |  |  | x |  |  |  |  |  |  |  |  |  |  |
| Tsartsali 2009 |  |  |  |  |  |  |  | x |  |  |  |  |  |  |  |  |  |  |
| Mirmiran 2007 |  |  |  |  |  |  |  | x |  |  |  |  |  |  |  |  |  |  |
| Venter 2010 |  |  |  |  |  |  |  | x |  |  |  |  |  |  |  |  |  |  |
| Gewa 2013 |  |  |  |  |  |  |  | x |  |  |  |  |  |  |  |  |  |  |
| Øverby 2012 |  |  |  |  |  |  |  | x |  |  |  |  |  |  |  |  |  |  |
| Leal 2011 |  |  |  |  |  |  |  | x |  |  |  |  |  |  |  |  |  |  |
| Osler 1993 |  |  |  |  |  |  |  | x |  |  |  |  |  |  |  |  |  |  |
| Chapman 1997 |  |  |  |  |  |  |  | x |  |  |  |  |  |  |  |  |  |  |
| Evans 2012 |  |  |  |  |  |  |  | x |  |  |  |  |  |  |  |  |  |  |
| Gans 1990 |  |  |  |  |  |  |  | x |  |  |  |  |  |  |  |  |  |  |
| Huang 2004 |  |  |  |  |  |  |  | x |  |  |  |  |  |  |  |  |  |  |
| Jarpe-Ratner 2016 |  |  |  |  |  |  |  | x |  |  |  |  |  |  |  |  |  |  |
| Larson 2006 |  |  |  |  |  |  |  | x |  |  |  |  |  |  |  |  |  |  |
| Laska 2012 |  |  |  |  |  |  |  | x |  |  |  |  |  |  |  |  |  |  |
| McAleese 2007 | 2 |  |  |  |  |  |  | x |  |  |  |  | x |  |  |  |  |  |
| Miller 2014 |  |  |  |  |  |  |  | x |  |  |  |  |  |  |  |  |  |  |
| Pirouznia 2001 |  |  |  |  |  |  |  | x |  |  |  |  |  |  |  |  |  |  |
| Schober 2016 |  |  |  |  |  |  |  | x |  |  |  |  |  |  |  |  |  |  |
| Trexler 1993 |  |  |  |  |  |  |  | x |  |  |  |  |  |  |  |  |  |  |
| Williams 2016 |  |  |  |  |  |  |  | x |  |  |  |  |  |  |  |  |  |  |
| Caraher 2013 |  |  |  |  |  |  |  | x |  |  |  |  |  |  |  |  |  |  |
| Ronto 2017 |  |  |  |  |  |  |  | x |  |  |  |  |  |  |  |  |  |  |
| Swaminathan 2009 |  |  |  |  |  |  |  | x |  |  |  |  |  |  |  |  |  |  |
| McKinley 2005 |  |  |  |  |  |  |  | x |  |  |  |  |  |  |  |  |  |  |
| Chatterjee 2016 |  |  |  |  |  |  |  | x |  |  |  |  |  |  |  |  |  |  |
| Lukas 2011 |  |  |  |  |  |  |  | x |  |  |  |  |  |  |  |  |  |  |
| Bohm 2015 |  |  |  |  |  |  |  | x |  |  |  |  |  |  |  |  |  |  |
| Bohm 2016 |  |  |  |  |  |  |  | x |  |  |  |  |  |  |  |  |  |  |
| Rees 2010 | 4 |  |  |  |  |  |  |  | x |  | x |  |  | x | x |  |  |  |
| Maes 2011 | 2 |  |  |  |  |  |  |  | x |  |  |  |  | x |  |  |  |  |
| Bech-Larsen 2013 | 2 |  |  |  |  |  |  |  | x |  |  |  |  | x |  |  |  |  |
| Räihä 2012 | 2 |  |  |  |  |  |  |  | x |  | x |  |  |  |  |  |  |  |
| Chamberland 2017 |  |  |  |  |  |  |  |  | x |  |  |  |  |  |  |  |  |  |
| Chung 2018 |  |  |  |  |  |  |  |  | x |  |  |  |  |  |  |  |  |  |
| Tumin 2016 |  |  |  |  |  |  |  |  | x |  |  |  |  |  |  |  |  |  |
| Nicklas 1998 | 2 |  |  |  |  |  |  |  |  | x |  |  |  |  | x |  |  |  |
| Bessems 2012 | 2 |  |  |  |  |  |  |  |  |  | x |  |  |  | x |  |  |  |
| Singh 2009 |  |  |  |  |  |  |  |  |  |  | x |  |  |  |  |  |  |  |
| van Nassau 2014 |  |  |  |  |  |  |  |  |  |  | x |  |  |  |  |  |  |  |
| Viggiano 2015 |  |  |  |  |  |  |  |  |  |  | x |  |  |  |  |  |  |  |
| Carfora 2016 |  |  |  |  |  |  |  |  |  |  | x |  |  |  |  |  |  |  |
| Ermitisi |  |  |  |  |  |  |  |  |  |  | x |  |  |  |  |  |  |  |
| Busch 2016 |  |  |  |  |  |  |  |  |  |  | x |  |  |  |  |  |  |  |
| Spence 2014 |  |  |  |  |  |  |  |  |  |  | x |  |  |  |  |  |  |  |
| Ensaff 2015 |  |  |  |  |  |  |  |  |  |  | x |  |  |  |  |  |  |  |
| Ardik 2016 |  |  |  |  |  |  |  |  |  |  | x |  |  |  |  |  |  |  |
| Kastorini 2012 |  |  |  |  |  |  |  |  |  |  | x |  |  |  |  |  |  |  |
| Sahingoz 2019 |  |  |  |  |  |  |  |  |  |  | x |  |  |  |  |  |  |  |
| Hovdenak 2019 |  |  |  |  |  |  |  |  |  |  | x |  |  |  |  |  |  |  |
| Souza 2019 |  |  |  |  |  |  |  |  |  |  | x |  |  |  |  |  |  |  |
| Sevil 2019 |  |  |  |  |  |  |  |  |  |  | x |  |  |  |  |  |  |  |
| Campos 2012 |  |  |  |  |  |  |  |  |  |  | x |  |  |  |  |  |  |  |
| Yusop 2018 |  |  |  |  |  |  |  |  |  |  |  | x |  |  |  |  |  |  |
| Toral 2012 | 2 |  |  |  |  |  |  |  |  |  |  | x |  |  |  |  |  | x |
| Lana 2013 |  |  |  |  |  |  |  |  |  |  |  | x |  |  |  |  |  |  |
| Jalambadani |  |  |  |  |  |  |  |  |  |  |  | x |  |  |  |  |  |  |
| Gur 2019 |  |  |  |  |  |  |  |  |  |  |  | x |  |  |  |  |  |  |
| Freen 2003 |  |  |  |  |  |  |  |  |  |  |  | x |  |  |  |  |  |  |
| Freen 2005 |  |  |  |  |  |  |  |  |  |  |  | x |  |  |  |  |  |  |
| Filgueiras 2018 |  |  |  |  |  |  |  |  |  |  |  | x |  |  |  |  |  |  |
| Di Noia 2008 |  |  |  |  |  |  |  |  |  |  |  | x |  |  |  |  |  |  |
| Boff 2018 |  |  |  |  |  |  |  |  |  |  |  | x |  |  |  |  |  |  |
| Bere 2007 |  |  |  |  |  |  |  |  |  |  |  |  | x |  |  |  |  |  |
| O’Neil 2002 |  |  |  |  |  |  |  |  |  |  |  |  | x |  |  |  |  |  |
| Gortmaker 1999 |  |  |  |  |  |  |  |  |  |  |  |  | x |  |  |  |  |  |
| Lubans 2012 |  |  |  |  |  |  |  |  |  |  |  |  |  | x |  |  |  |  |
| Sharma 2015 |  |  |  |  |  |  |  |  |  |  |  |  |  | x |  |  |  |  |
| Thompson 2009 |  |  |  |  |  |  |  |  |  |  |  |  |  | x |  |  |  |  |
| Baños 2012 |  |  |  |  |  |  |  |  |  |  |  |  |  | x |  |  |  |  |
| Amani 2006 | 2 |  |  |  |  |  |  |  |  |  |  |  |  |  | x |  |  | x |
| Amaro 2006 |  |  |  |  |  |  |  |  |  |  |  |  |  |  | x |  |  |  |
| Bjelland 2015 |  |  |  |  |  |  |  |  |  |  |  |  |  |  | x |  |  |  |
| Cunha 2013 | 2 |  |  |  |  |  |  |  |  |  |  |  |  |  | x |  |  | x |
| Forneris 2010 |  |  |  |  |  |  |  |  |  |  |  |  |  |  | x |  |  |  |
| Francis 2010 |  |  |  |  |  |  |  |  |  |  |  |  |  |  | x |  |  |  |
| Ghaffari 2019 |  |  |  |  |  |  |  |  |  |  |  |  |  |  | x |  |  |  |
| Gray 2015 | 2 |  |  |  |  |  |  |  |  |  |  |  |  |  | x |  | x |  |
| Ickovics 2019 |  |  |  |  |  |  |  |  |  |  |  |  |  |  | x |  |  |  |
| Ochoa 2017 |  |  |  |  |  |  |  |  |  |  |  |  |  |  | x |  |  |  |
| Anderson 2005 |  |  |  |  |  |  |  |  |  |  |  |  |  |  |  | x |  |  |
| Angelopoulos 2009 |  |  |  |  |  |  |  |  |  |  |  |  |  |  |  | x |  |  |
| Beaulieu 2012 |  |  |  |  |  |  |  |  |  |  |  |  |  |  |  | x |  |  |
| Jemmott 2011 |  |  |  |  |  |  |  |  |  |  |  |  |  |  |  | x |  |  |
| Karimi 2013 | 2 |  |  |  |  |  |  |  |  |  |  |  |  |  |  | x |  | x |
| Kothe 2011 |  |  |  |  |  |  |  |  |  |  |  |  |  |  |  | x |  |  |
| Kothe 2012 |  |  |  |  |  |  |  |  |  |  |  |  |  |  |  | x |  |  |
| Prelip 2018 |  |  |  |  |  |  |  |  |  |  |  |  |  |  |  | x |  |  |
| Spiegel 2006 |  |  |  |  |  |  |  |  |  |  |  |  |  |  |  | x |  |  |
| Arlinghaus 2017 |  |  |  |  |  |  |  |  |  |  |  |  |  |  |  |  | x |  |
| Baskin 2009 |  |  |  |  |  |  |  |  |  |  |  |  |  |  |  |  | x |  |
| Contento 2007 |  |  |  |  |  |  |  |  |  |  |  |  |  |  |  |  | x |  |
| Gray 2016 |  |  |  |  |  |  |  |  |  |  |  |  |  |  |  |  | x |  |
| Lee 2013 |  |  |  |  |  |  |  |  |  |  |  |  |  |  |  |  | x |  |
| Duncan 2019 |  |  |  |  |  |  |  |  |  |  |  |  |  |  |  |  | x |  |
| Fahlman 2008 |  |  |  |  |  |  |  |  |  |  |  |  |  |  |  |  | x |  |
| Fairclough 2013 |  |  |  |  |  |  |  |  |  |  |  |  |  |  |  |  | x |  |
| Heo 2018 |  |  |  |  |  |  |  |  |  |  |  |  |  |  |  |  | x |  |
| Irwin 2012 |  |  |  |  |  |  |  |  |  |  |  |  |  |  |  |  | x |  |
| Irwin 2010 |  |  |  |  |  |  |  |  |  |  |  |  |  |  |  |  | x |  |
| Kipping 2014 |  |  |  |  |  |  |  |  |  |  |  |  |  |  |  |  | x |  |
| Lawlor 2011 |  |  |  |  |  |  |  |  |  |  |  |  |  |  |  |  | x |  |
| Koch 2019 |  |  |  |  |  |  |  |  |  |  |  |  |  |  |  |  | x |  |
| Lepe 2019 |  |  |  |  |  |  |  |  |  |  |  |  |  |  |  |  | x |  |
| Li 2010 |  |  |  |  |  |  |  |  |  |  |  |  |  |  |  |  | x |  |
| Xu 2017 |  |  |  |  |  |  |  |  |  |  |  |  |  |  |  |  | x |  |
| Olivares 2005 |  |  |  |  |  |  |  |  |  |  |  |  |  |  |  |  | x |  |
| Tsai 2009 |  |  |  |  |  |  |  |  |  |  |  |  |  |  |  |  | x |  |
| Tucker 2015 |  |  |  |  |  |  |  |  |  |  |  |  |  |  |  |  | x |  |
| Zhou 2019 |  |  |  |  |  |  |  |  |  |  |  |  |  |  |  |  | x |  |
| Zhou 2018 |  |  |  |  |  |  |  |  |  |  |  |  |  |  |  |  | x |  |
| Stock 2007 |  |  |  |  |  |  |  |  |  |  |  |  |  |  |  |  | x |  |
| El Rayess 2017 |  |  |  |  |  |  |  |  |  |  |  |  |  |  |  |  | x |  |
| Gittelsohn 2013 |  |  |  |  |  |  |  |  |  |  |  |  |  |  |  |  | x |  |
| Kohlstatdt 2016 |  |  |  |  |  |  |  |  |  |  |  |  |  |  |  |  | x |  |
| Kohlstatdt 2015 |  |  |  |  |  |  |  |  |  |  |  |  |  |  |  |  | x |  |
| Linton 2014 |  |  |  |  |  |  |  |  |  |  |  |  |  |  |  |  | x |  |
| Luesse 2019 |  |  |  |  |  |  |  |  |  |  |  |  |  |  |  |  | x |  |
| Molaison 2005 |  |  |  |  |  |  |  |  |  |  |  |  |  |  |  |  | x |  |
| Wright 2012 |  |  |  |  |  |  |  |  |  |  |  |  |  |  |  |  | x |  |
| Gittelsohn 2014 |  |  |  |  |  |  |  |  |  |  |  |  |  |  |  |  | x |  |
| Sato 2016 |  |  |  |  |  |  |  |  |  |  |  |  |  |  |  |  | x |  |
| Trude 2018 |  |  |  |  |  |  |  |  |  |  |  |  |  |  |  |  | x |  |
| Steeves 20198 |  |  |  |  |  |  |  |  |  |  |  |  |  |  |  |  | x |  |
| Saez 2018 |  |  |  |  |  |  |  |  |  |  |  |  |  |  |  |  | x |  |
| Bell 2017 |  |  |  |  |  |  |  |  |  |  |  |  |  |  |  |  | x |  |
| Bogart 2014 |  |  |  |  |  |  |  |  |  |  |  |  |  |  |  |  | x |  |
| Bogart 2016 |  |  |  |  |  |  |  |  |  |  |  |  |  |  |  |  | x |  |
| Franken 2018 |  |  |  |  |  |  |  |  |  |  |  |  |  |  |  |  | x |  |
| Jackson 2010 |  |  |  |  |  |  |  |  |  |  |  |  |  |  |  |  | x |  |
| Leung 2017 |  |  |  |  |  |  |  |  |  |  |  |  |  |  |  |  | x |  |
| Necheles 2007 |  |  |  |  |  |  |  |  |  |  |  |  |  |  |  |  | x |  |
| Smit 2016 |  |  |  |  |  |  |  |  |  |  |  |  |  |  |  |  | x |  |
| Tamiru 2016 |  |  |  |  |  |  |  |  |  |  |  |  |  |  |  |  | x |  |
| Akdemir 2017 |  |  |  |  |  |  |  |  |  |  |  |  |  |  |  |  |  | x |
| Chagas 2020 |  |  |  |  |  |  |  |  |  |  |  |  |  |  |  |  |  | x |
| Brito 2019 |  |  |  |  |  |  |  |  |  |  |  |  |  |  |  |  |  | x |
| Fonseca 2019 |  |  |  |  |  |  |  |  |  |  |  |  |  |  |  |  |  | x |
| Keshani 2019 |  |  |  |  |  |  |  |  |  |  |  |  |  |  |  |  |  | x |
| Leventhal 2016 |  |  |  |  |  |  |  |  |  |  |  |  |  |  |  |  |  | x |
| Lin 2017 |  |  |  |  |  |  |  |  |  |  |  |  |  |  |  |  |  | x |
| Najimi 2013 |  |  |  |  |  |  |  |  |  |  |  |  |  |  |  |  |  | x |
| Sichieri 2009 |  |  |  |  |  |  |  |  |  |  |  |  |  |  |  |  |  | x |
| Sichieri 2013 |  |  |  |  |  |  |  |  |  |  |  |  |  |  |  |  |  | x |
| D. Wang 2013 |  |  |  |  |  |  |  |  |  |  |  |  |  |  |  |  |  | x |
| D. Wang 2014 |  |  |  |  |  |  |  |  |  |  |  |  |  |  |  |  |  | x |
| Stewart 2015 |  |  |  |  |  |  |  |  |  |  |  |  |  |  |  |  |  | x |
| Yusoff 2012 |  |  |  |  |  |  |  |  |  |  |  |  |  |  |  |  |  | x |
| Yusoff 2013 |  |  |  |  |  |  |  |  |  |  |  |  |  |  |  |  |  | x |
| Dansa 2019 |  |  |  |  |  |  |  |  |  |  |  |  |  |  |  |  |  | x |
| Brito 2015 |  |  |  |  |  |  |  |  |  |  |  |  |  |  |  |  |  | x |
| Ghrayeb 2013 |  |  |  |  |  |  |  |  |  |  |  |  |  |  |  |  |  | x |
| Hosseini 2015 |  |  |  |  |  |  |  |  |  |  |  |  |  |  |  |  |  | x |
| Sharif 2020 |  |  |  |  |  |  |  |  |  |  |  |  |  |  |  |  |  | x |
| Shen 2020 |  |  |  |  |  |  |  |  |  |  |  |  |  |  |  |  |  | x |
| Taghdisi 2016 |  |  |  |  |  |  |  |  |  |  |  |  |  |  |  |  |  | x |
