# Supplementary material for: School-based healthy eating interventions for adolescents aged 10–19 years: an umbrella review
Source: Int J Behav Nutr Phys Act. 2024 Oct 14;21:117. doi: 10.1186/s12966-024-01668-6 (PMC11472496; doi:10.1186/s12966-024-01668-6)
Supplement: Supplementary file 4 — Supplementary Material 4 [file 12966_2024_1668_MOESM4_ESM.docx]

Supplementary file

Table: JBI critical appraisal checklist

| **Author** | **Year** | **Q1** | **Q2** | **Q3** | **Q4** | **Q5** | **Q6** | **Q7** | **Q8** | **Q9** | **Q10** | **Q11** | **Total** | **Quality** |
| --- | --- | --- | --- | --- | --- | --- | --- | --- | --- | --- | --- | --- | --- | --- |
| Alcântara CMD et al. | 2018 | y | y | y | y | n | n | n | n/a | n | n | n | 4 | medium |
| Bailey CJ et al. | 2019 | y | y | y | y | y | y | y | n/a | n | n | y | 8 | high |
| Calvert S et al. | 2019 | y | y | y | y | y | y | y | n/a | n | n | y | 8 | high |
| Champion KE et al. | 2019 | y | y | y | y | y | y | y | y | n | n | y | 9 | high |
| Hackman et al | 2014 | y | y | y | y | n | y | n | n/a | n | y | y | 8 | high |
| McHugh C et al. | 2020 | y | y | y | y | y | y | y | n/a | n | n | y | 8 | high |
| Medeiros et al | 2022 | y | y | y | y | y | y | y | y | y | y | y | 11 | high |
| Meiklejohn et al. | 2016 | y | y | y | y | y | y | y | n/a | n | y | y | 9 | high |
| Melo et al. | 2017 | y | y | y | y | y | y | y | n/a | n | n | y | 9 | high |
| Nakabayashi J et al. | 2020 | y | n | y | y | y | y | y | n/a | n | n | y | 7 | medium |
| Pierre CS er al | 2021 | y | y | y | y | y | y | y | n/a | n | n | y | 9 | high |
| Rose K et al. | 2021 | y | y | y | y | y | y | y | n/a | n | y | y | 10 | high |
| Sa JD & Lock K | 2008 | y | y | y | y | y | y | n | n/a | n | y | y | 9 | high |
| Shinde | 2023 | y | y | y | y | y | y | y | n/a | n | y | y | 9 | high |
| Tallon JM et al. | 2019 | y | y | y | y | y | n | n | n/a | n | n | y | 6 | medium |
| Van Cauwenberghe et al. | 2010 | y | y | y | y | y | y | n | n/a | n | y | y | 8 | high |
| Vézina-Im LA et al. | 2017 | y | y | y | y | y | y | y | n/a | n | y | y | 9 | high |

Questions

1. Were the criteria for inclusion in the sample clearly defined?

2. Were the study subjects and the setting described in detail?

3. Was the exposure measured in a valid and reliable way?

4. Were objective, standard criteria used for measurement of the condition?

5. Were confounding factors identified?

6. Were strategies to deal with confounding factors stated?

7. Were the outcomes measured in a valid and reliable way?

8. Was appropriate statistical analysis used?

9. Was the likelihood of bias assessed?

10. Were recommendations for policy and/or practice supported by reported data?

11. Were the specific directives for new research appropriate?
