# Supplementary material for: School-based healthy eating interventions for adolescents aged 10–19 years: an umbrella review
Source: Int J Behav Nutr Phys Act. 2024 Oct 14;21:117. doi: 10.1186/s12966-024-01668-6 (PMC11472496; doi:10.1186/s12966-024-01668-6)
Supplement: Supplementary file 5 — Supplementary Material 5 [file 12966_2024_1668_MOESM5_ESM.docx]

**Table 1:** Categorisation of healthy eating education components using behaviour change technique taxonomy

| **Author, year** | **Feedback and monitoring:** tracking healthy eating include digital and non-digital methods, using self-assessment and external monitoring^a^ | **Shaping knowledge:** instructions and experiments to teach and reinforce the adoption and maintenance of healthy eating behaviours^b^ | **Associations:** introducing stimuli with the purpose of cueing healthy eating behaviour^c^ | **Effectiveness, methodogical quality** |
| --- | --- | --- | --- | --- |
| Alcântara 2018 (26) |  |  |  | Likely effective; low quality |
| Melo GRDA 2017 (38) |  |  |  | Likely effective; high quality |
| Tallon JM 2019 (30) |  |  |  | Likely effective; moderate quality |

**Footnote:** a: apps (to measure and monitor daily food intake), SMS-based diaries (sending timed text prompts for users to report food intake, enabling real-time tracking and feedback on eating behaviour); b: workshops, games, blogs; c: virtual canteen (digital simulation that mimics the experience of selecting and consuming meals in a canteen setting), SMS

**Table 2** Categorisation of multi-components using behaviour change technique taxonomy

| **Author, year** | **Feedback and monitoring:** tracking healthy eating include digital and non-digital methods, using self-assessment and external monitoring^a^ | **Social support:** Peer support to encourage healthy eating^b^ | **Shaping knowledge:** instructions and experiments to teach and reinforce the adoption and maintenance of healthy eating behaviours^c^ | **Associations:** introducing stimuli with the purpose of cueing healthy eating behaviour^d^ | **Rewards: r**ewards for purchasing healthy foods and maintaining healthy eating behaviour^e^ | **Antecedents:** restructuring the physical and social environments in school to facilitate healthy eating behaviour^f^ | **Effectiveness, methodogical quality** |
| --- | --- | --- | --- | --- | --- | --- | --- |
| Bailey CJ 2019 (33) |  |  |  |  |  |  | Promising; high quality |
| Calvert S 2019; (34) |  |  |  |  |  |  | Promising; high quality |
| Champion KE 2019 (41) |  |  |  |  |  |  | No conclusion, high quality |
| Hackman 2014 (35) |  |  |  |  |  |  | Promising; high quality |
| McHugh C 2020 (36) |  |  |  |  |  |  | No conclusion; high quality |
| Medeiros 2022 (42) |  |  |  |  |  |  | Promising; high quality |
| Meiklejohn 2016 (37) |  |  |  |  |  |  | No conclusion; high quality |
| Nakabayashi J 2020 (39) |  |  |  |  |  |  | Promising; moderate quality |
| Pierre CS 2021 (40) |  |  |  |  |  |  | Likely effective; high quality |
| Rose K 2021 (28) |  |  |  |  |  |  | Likely effective; high quality |
| Sa JD & Lock K, 2008 (29) |  |  |  |  |  |  | Promising; high quality |
| Shinde 2023 (27) |  |  |  |  |  |  | Promising; high quality |
| Van 2010 (31) |  |  |  |  |  |  | No conclusion; high quality |
| Vézina-Im 2017 (32) |  |  |  |  |  |  | Promising; high quality |

**Footnote:** a: quiz, self-assessment and evaluation diary; b: parents and family involvement to share experience, challenges and encourage healthy eating behaviour; c: workshops, conference, campaign, cooking classes, games, field visits, blog by health coach for students and workshop, healthy eating information handouts and homework for parents; d: nutri-advice kiosk (stand-alone booth that provides nutrition-related information and personalised advice), entertainments (media shows, drama, puppet shows, visits by inspiring personalities), posters, SMS, emails, counselling via mHealth (nutritional behavioural counselling), healthy eating club, ; e: rewards to adolescents, for healthy eating behaviour, and loyalty programme, coupon for parents); f: school FV gardening, school food marketing, training for kitchen staff, canteen modification, such as increased availability of healthy foods, reduced fruit prices, involving parents in the school nutrition council group, parents’ meeting, invite to school meals, free healthy foods to parents
